# UTX deletion promotes M2 macrophage polarization by epigenetically regulating endothelial cell-macrophage crosstalk after spinal cord injury

**DOI:** 10.1186/s12951-023-01986-0

**Published:** 2023-07-15

**Authors:** Wei Peng, Yong Xie, Zixiang Luo, Yudong Liu, Jiaqi Xu, Chengjun Li, Tian Qin, Hongbin Lu, Jianzhong Hu

**Affiliations:** 1 Department of Spine Surgery and Orthopaedics, Xiangya Hospital, Central South University, Changsha, China; 2 Department of Sports Medicine, Xiangya Hospital, Central South University, Changsha, China; 3 Key Laboratory of Organ Injury, Aging and Regenerative Medicine of Hunan Province, Changsha, China; 4 Hunan Engineering Research Center of Sports and Health, Changsha, China; 5 National Clinical Research Center for Geriatric Disorders, Xiangya Hospital, Central South University, Changsha, China; 6 Department of Spine Surgery, Wuxi 9th Affiliated Hospital of Soochow University, Wuxi, China

**Keywords:** Epigenetics, Exosomes, Macrophage polarization, Spinal cord injury, UTX

## Abstract

Macrophages polarized to the M2 subtype after spinal cord injury (SCI) are beneficial for promoting neurological recovery. The crosstalk between endothelial cells (ECs) and macrophages is crucial for the imbalance between proinflammatory and pro-resolving responses caused by macrophage heterogeneity; however, this crosstalk is strengthened post-SCI, leading to inflammatory cascades and second damage. As a powerful means to regulate gene expression, epigenetic regulation of the interaction between immune cells and ECs in SCI is still largely unknown. Our previous research demonstrated that the histone demethylase UTX deletion in ECs (UTX^−/−^ ECs) promotes neurological recovery, while the precise mechanism is unrevealed. Here, we discovered that UTX^−/−^ ECs polarize macrophages toward the M2 subtype post-SCI. Macrophage deficiency could block the neurological recovery caused by the knockdown of UTX. The exosomes from UTX^−/−^ ECs mediate this crosstalk. In addition, we found UTX, H3K27, and miR-467b-3p/Sfmbt2 promoters forming a regulatory complex that upregulates the miR-467b-3p in UTX^−/−^ ECs. And then, miR-467b-3p transfers to macrophages by exosomes and activates the PI3K/AKT/mTOR signaling by decreasing PTEN expression, finally polarizing macrophage to the M2 subtype. This study reveals a mechanism by epigenetic regulation of ECs-macrophages crosstalk and identifies potential targets, which may provide opportunities for treating SCI.

**Graphical Abstract:**

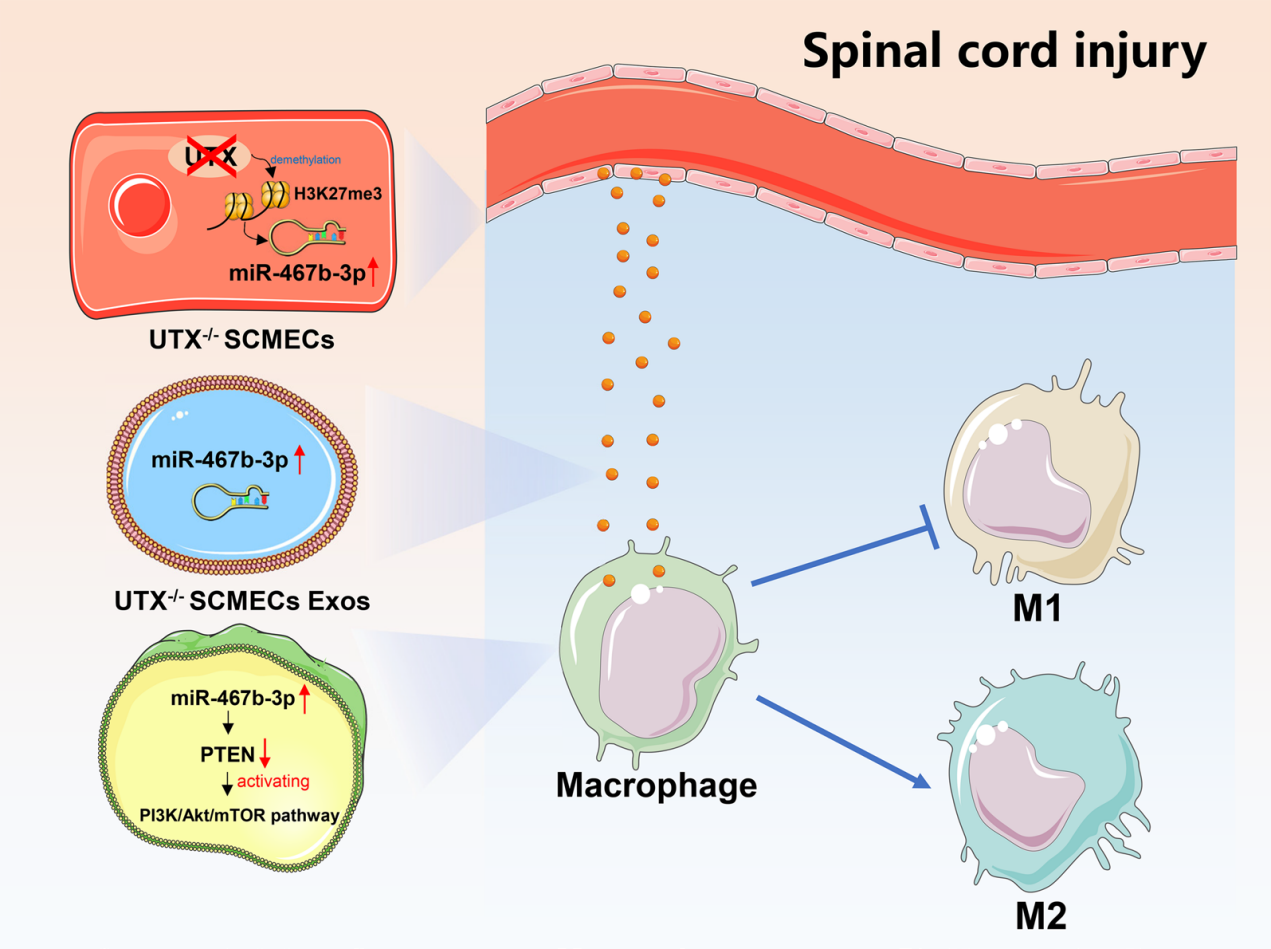

**Supplementary Information:**

The online version contains supplementary material available at 10.1186/s12951-023-01986-0.

## Introduction

Spinal cord injury (SCI) is a severe traumatic disease of the central nervous system (CNS), resulting in damage to motor, sensory, and autonomic functions. According to the latest global epidemiological studies, there are currently 27 million people with SCI worldwide, with a prevalence of 25–31 per million and about 15 million new cases yearly [1, 2]. A lack of understanding of the physiological and pathological mechanisms following SCI limits the development of effective treatment strategies [3].

Macrophages play a significant role in regulating neuroinflammation and tissue repair [4–6]. They are known to have highly plastic in response to varying environments, exhibiting different phenotypes ranging from the classically activated, pro­inflammatory M1 to alternatively activated, anti-inflammatory M2 [5–7]. The injured spinal tissue is dominated by the M1 macrophage population during SCI, which produces high levels of pro-inflammatory cytokines and exacerbates acute injury, harming nervous tissue regeneration. Meanwhile, M2 macrophage protects neighboring cells by removing cell debris and releasing trophic factors for vascular regeneration, cell proliferation, and tissue growth, which is advantageous for SCI repair [8–10]. The phenotype polarization of macrophages is likely dependent on their activation status, and balancing this polarization is a promising approach to treating SCI.

Endothelial cells (ECs) could dynamically regulate themselves in response to the extracellular microenvironment promptly [11]. They can directly impact the immune response process, affecting tissue regeneration and repair [11]. In vascular-immune regulation, ECs primarily communicate with macrophages, which release cytokines jointly with immune cells and activate or inhibit immune function synergistically [12, 13]. The crosstalk between ECs and macrophages is crucial for the imbalance between proinflammatory and pro-resolving responses caused by macrophage heterogeneity; however, this crosstalk is strengthened post-SCI, leading to inflammatory cascades and second damage [13, 14]. According to recent studies, ECs can communicate with mature macrophages through tight junctions, instructing macrophages to polarize towards M2 macrophages [15].

Exosomes, vesicles with 40–150 nm diameters, contribute to intercellular communication and have become a hotspot for regenerative medicine research [16]. The exosomal non-coding RNAs and proteins activate the signaling pathways in the recipient cells, affecting the cell's biological function [17, 18]. The exosomal miRNAs can mediate the EC-macrophage crosstalk that reduces the inflammatory response [13, 19]. In recent studies, exosomes derived from ECs containing miR-223 and miR-27b-3p regulate macrophage phenotypes, resulting in immune homeostasis [20].

The epigenetic regulation of ECs has provided new insights into the regulation of plasticity in ECs [21, 22]. Histone demethylase UTX/KDM6A removes methyl groups from H3K27me3/me2, thus initiating transcription of target genes. A loss of H3K27me3 imprinting can upregulate the expression of clustered miRNAs according to transcriptome and DNA methylation analyses [23]. In our previous study, UTX increases miR-24 levels to inhibit NeuroD1's effects on axonal regeneration and neurological recovery after SCI [24]. Previously, we also found that the knockdown of UTX (UTX-KO) in Tek^+^ ECs can significantly promote vascular regeneration after SCI, thereby facilitating neurological recovery [25]. However, the specific mechanism of UTX deletion in Tek^+^ ECs promoting the neurological recovery post-SCI has not been fully elucidated.

This study aims to identify the role of the exosomes derived from UTX-KO ECs in mediating EC-macrophage crosstalk and regulating macrophage polarization after SCI. By combining miRNA sequencing analysis with gene KO experiments, we demonstrate that UTX deletion upregulates the expression of miR-467b-3p, which enters the macrophage by exosomes to inhibit PTEN’s transcription, activating the PI3K/AKT/mTOR signaling pathway. This study is expected to reveal new mechanisms of macrophage polarization regulation by ECs and identify new regulatory targets, which may provide a new theoretical basis for treating SCI.

## Materials and methods

### Mice

All animal experiments were approved by the guidelines of the Ethics Committee of Central South University (CSU), complying with the guide for the care and use of laboratory animals (NIH Publications No. 8023, revised 1978). We purchased C57BL/6J (Wide Type, WT) female mice from Charles River. The UTX^flox/flox^ (stock no. 021926) mouse strain was purchased from Jackson Laboratory, and Tek^Cre^ (SJ-008863) mouse strain was from Shanghai Model Organisms. Crossing heterozygous Tek^Cre^ mice with pure UTX^flox/flox^ mice, we obtained the following genotypes for further studies: UTX^flox/flox^ (UTX^f/f^) and Tek^Cre^; UTX^flox/flox^ (UTX^−/−^). All mice were housed in a specific pathogen-free facility with free access to diet and water in a 12-h day/night cycle.

### Macrophages depletion model and contusion SCI model

A mouse model of macrophage depletion was constructed by continuous intravenous injection of chlorophosphate liposomes three days before surgery and every other day after the operation. The control group used blank liposomes. An SCI model of female mice was established, as previously described [25]. In brief, after profound anesthesia (ketamine/xylazine/acepromazine), laminectomy exposed the spinal cord in the mice's 10th thoracic vertebra and then constructed the SCI model using Allen's weight-drop apparatus. The sham model was performed only with a laminectomy to expose the spinal cord without SCI.

### Isolation, culture, and identification of spinal cord ECs

Primary spinal cord ECs were isolated, as our previous study described [26]. In brief, the spinal cord was obtained, then minced until a milky suspension. After centrifuging, the supernatant was removed. Following 0.1% (v/v), collagenase type II (Sigma, USA) was added to digest for 1 h, and the residue was collected after centrifuge. To remove the residue’s myelin, 20 ml of BSA-DMEM (20%, w/v) was added. Following the upper myelin discarded, 0.1% (v/v) collagenase/dispase (Sigma, USA) was used to digest into single cells. After centrifuging and discarding the supernatant, cells were seeded into cell culture plates for the next experiment.

### Isolation and identification of primary bone marrow macrophages (BMDMs)

Primary BMDMs were isolated, as in our previous study [26]. Immunofluorescence and flow cytometry was used to detect the macrophage surface markers CD11b (PerCP-Cy5.5, Biolegend) and F4/80 (FITC, Biolegend) to identify macrophage purity.

### Isolation and identification of the exosomes derived from ECs

The UTX^f/f^ ECs-derived exosomes (UTX^f/f^-Exos) and UTX^−/−^ ECs-derived exosomes (UTX^−/−^-Exos) were isolated by differential centrifugation, as reported in our previous studies [27]. Transmission electron microscopy (TEM; Hitachi, JPN) was used to identify the morphology of exosomes. Nanoparticle tracking analysis (NTA) was used to measure exosome diameter and particle number. Western blotting was used to detect the characteristic markers of exosomes Calnexin, TSG101 and CD63 (1:1000, Abcam, USA).

### Dil-labeled ECs-Exos

ECs-derived exosomes (ECs-Exos) were labeled with a red fluorescent lipophilic dye Dil (Sigma, USA) to monitor the motion of the exosomes. In brief, after ECs-Exos were incubated with 5 µM Dil for 15 min, the labeled exosomes were washed twice and resuspended in sterile PBS. Then, they could be used for subsequent in vivo and in vitro experiments.

### Establishment of an EC-macrophage co-cultivation system

This study used a 12-well plate with 1 μm Transwell (Corning, USA) to establish a co-culture system for ECs and macrophages. The ECs were resuspended and diluted to 2 × 10^5^ cells/ml with a complete medium and seeded in the upper chamber of the transwell. Macrophages were prepared similarly and seeded in the lower chamber to construct the EC-macrophage co-culture system.

### Evaluation of the locomotive function

The BMS (Basso Mouse Scale) was utilized before surgery and 1, 3, 7, 10, 14, 21, and 28 days after SCI to evaluate the motor function [28]. A BMS sub-scoring system was also assessed as a supplement to avoid bias in the BMS scoring data. Each mouse was observed for 5 min, and the average BMS and sub-scoring were recorded by two trained researchers and blinded to the experimental design.

### Neuroelectrophysiology

To assess the hindlimbs of movement recovery after SCI, the MEPs (motor evoked potentials) were recorded by electromyography conducted 4 weeks post-SCI as described in our previous research [29]. In brief, after the mice were anesthetized, the stimulating electrode was set to the surface of the skull corresponding to the cortical motor area. At the same time, the recording electrode was inserted into the anterior tibial muscle of the contralateral hind limb. The reference electrode was placed in the subcutaneous tissue between the stimulating and recording electrodes. Ten consecutive electrical stimulations were performed at 1 min intervals to obtain mean MEP values (amplitude and latency).

### Lentiviral transfection

The lentiviral overexpression vector of UTX (pLV[Exp]-EGFP:T2A: Puro-EF1A > mCherry) and negative control were purchased from Haixing Biology Technology Co., Ltd. (Suzhou, China). The inhibitor, mimic, and negative control of miRNAs were purchased from RiboBio (Guangzhou, China). Cells were evenly grown in 6-well culture plates, and transfection experiments could be performed when the cells grew to a confluence of about 60–80%. Cell transfection was conducted following the manufacturer’s instructions.

### microRNA sequencing analysis and target gene prediction

High-throughput sequencing was performed using the Illumina HiSeq 2500 sequencing platform. Predicted target genes for miR-467b-5p were predicted using TargetScanMouse7.1 (https://www.targetscan.org/vert_71/) and miRWalk public databases (http://mirwalk.umm.uni-heidelberg.de/), and the intersection of the two predictions was analyzed by Venny 2.1 software. The intersection of the two databases was bioinformatical analysis using the g: Profile online website https://biit.cs.ut.ee/gprofiler/page/news, including GO functional annotation, KEGG pathway annotation, enrichment analysis, clustering analysis, and miRNA interaction network analysis.

### Dual-luciferase reporter assay

The binding site of miR-467b-3p to PTEN mRNA (mPTEN) (NM_008960.2 3) was found by the TargetScan database. The potential target pmiR-RB-ReportTM-mPTEN 3-UTR luciferase reporter plasmid was constructed by RiboBio (Guangzhou, China). The sea kidney luciferase gene (hRluc) was a reporter gene, and the firefly luciferase gene (hLuc) was an internal reference gene. The relative fluorescence intensity of hRluc luciferase was detected to identify whether the miR-467b-3p targeted regulates the expression of PTEN. The dual-Luciferase reporter test was performed using Promega's Dual-Luciferase® Reporter Assay System kit (Promega, USA) and following the manufacturer’s instructions.

### Immunofluorescence

Tissue or cell samples were incubated with 0.5% TritonX-100 in PBS for 30 min at room temperature and then closed with 5% BSA in PBS for 1 h. Next, the primary antibody should be incubated overnight at 4 °C. The next day, after washing with PBS, the sections were incubated with the secondary antibody for 1.5 h. Finally, the sections were mounted with Fluoroshield™ (GeneTex Inc) containing DAPI. Analysis was carried out by fluorescence microscopy (Zeiss Apotome 2, JPN). The results of the analysis were quantified using ImageJ software. Detailed information on antibodies was provided in Additional file 1: Table S1.

### Western blotting

Total protein was extracted by lysis in RIPA (Sigma, USA) lysis buffer. After measuring the protein concentration, equal amounts of protein were subjected to sodium dodecyl sulfate–polyacrylamide gel electrophoresis (SDS-PAGE) and transferred to polyvinylidene difluoride (PVDF) (Millipore, Billerica, MA) membranes. The membranes were closed in 5% skimmed milk and then incubated overnight at 4 °C with primary antibodies, followed by secondary antibodies. Finally, data were checked by a ChemiDoc XRS Plus luminescence image analyzer (Bio-Rad, USA) and enhanced chemiluminescence reagent (MIKX, China). Detailed information on antibodies was provided in Additional file 1: Table S1.

### Quantitative real-time PCR (qRT-PCR) analysis

Total RNA was extracted from tissues and cells using TRIzol reagent (Invitrogen, USA) and then reverse transcribed using the GoScript™ Reverse Transcription System reverse transcription kit (Promega, USA). Real-time fluorescence qPCR was performed using the GoScript™ qPCR Master Mix kit (Promega, USA) according to the instructions. GAPDH was used as an internal reference, and the corresponding gene expression was calculated using the 2-ΔΔCT method. All primer sequences used for qRT-PCR were provided in Additional file 1: Table S2. And all miRNA mimic/inhibitor sequences were in Additional file 1: Table S3.

### Statistical analysis

The results were statistically analyzed with SPSS 22.0 (SPSS, Inc.). All data were presented as the means ± standard deviation (SD). Statistical analysis of multiple-group comparison was performed by one-way analysis of variance (ANOVA), followed by the Bonferroni post hoc test. Values of *p* less than 0.05 were considered statistically significant.

## Result

### Macrophage deficiency could block the neurological recovery caused by the knockdown of UTX post-SCI

**Fig. 1:**
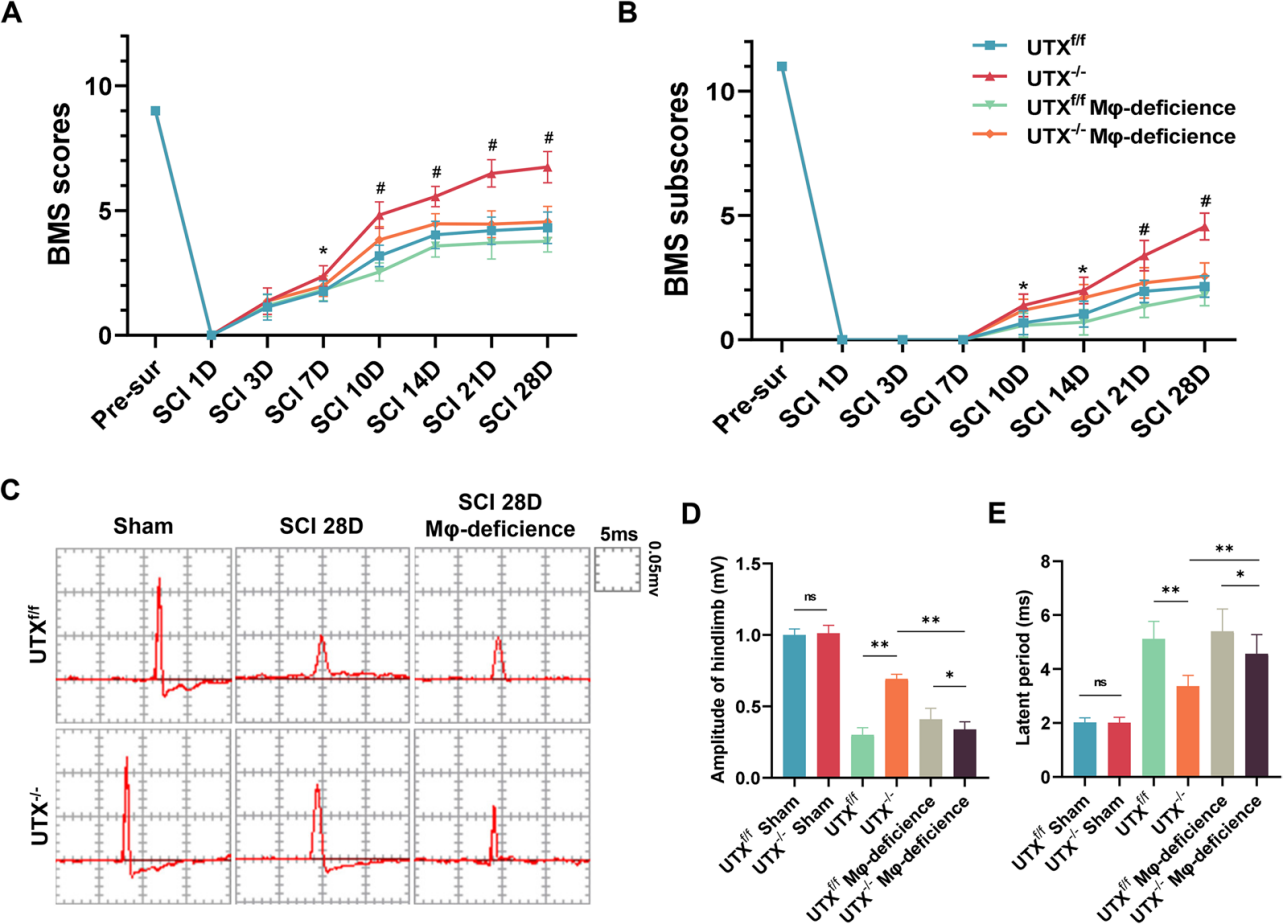
Macrophage depletion in UTX^−/−^ mice blocked neurological recovery post-SCI. **A**, **B** Distribution of the BMS scores and sub-scores over time post-SCI in UTX^f/f^, UTX^−/−^, UTX^f/f^ macrophage-deficient, and UTX^−/−^ macrophage-deficient groups. **C** Neuroelectrophysiology of macrophage deficiency in sham, UTX^f/f^ mice and UTX^−/−^ mice at 28 days after SCI. **D**, **E** Statistical analysis of amplitude and latency periods in **C**. ^ns^*P* > 0.05, *P < 0.05, **P < 0.01 vs. corresponding control mice. n = 5/per group

Our previous study showed that UTX-KO in Tek^+^ ECs can promote functional recovery after SCI; however, the exact mechanism has not been clarified [25]. As an epigenetic regulator, UTX has extensive effects through demethylating H3K27 histones, and mounting evidence suggests extensive communication between macrophages and ECs [11]. To elucidate the role of the macrophages in UTX-KO mice in functional recovery post-SCI, we knocked out UTX specifically in Tek^+^ ECs by crossbreeding Tek-Cre mice with UTX floxed mice (TekCre; UTX^flox/flox^, UTX^−/−^ mice) and established a macrophage model of UTX^−/−^ mice. To verify whether the macrophages in the injured region were depleted successfully, we first performed immunofluorescence to assess the effect of clodronate liposome. The results reveal that compared with the sham mice, the mice treated with PBS liposomes had an enormous number of F4/80^+^ macrophages in the injured epicenter and Iba-1^+^ microglia around them at 14 days after SCI, while the clodronate liposome treatment mice have a significant reduction of F4/80^+^ in the injured region and no significant change of Iba-1^+^ microglia (Additional file 1: Fig. S1), which indicated that we successfully constructed a mouse model of macrophage deficiency. Furthermore, we did the hindlimb motor function test, demonstrating that UTX-KO in Tek^+^ ECs improved the BMS scores and sub-scores starting at 7 days post-SCI till 28 days compared with UTX^f/f^ mice. These data indicated that UTX^−/−^ mice have more vital functional recovery ability than UTX^f/f^ mice. However, when the macrophages in the injured epicenter were depleted, the increased BMS scores and subs-cores of UTX^−/−^ mice were blocked (Fig. 1A and B). In addition, consistent with the BMS scores and sub-scores results, the hindlimb electrophysiological analysis showed that UTX^−/−^ mice exhibited a much higher amplitude of motor evoked potentials (MEPs) and a significantly shorter latent period compared to UTX^f/f^ mice at 28 days post-SCI; however, the macrophages in the epicenter of the injured region depleted can block this increase (Fig. 1C–E). These findings imply that macrophages are critical for promoting neurological recovery caused by UTX deletion in ECs post-SCI.

### The UTX^−/−^ ECs promote macrophages polarization toward M2 subtype after SCI

**Fig. 2:**
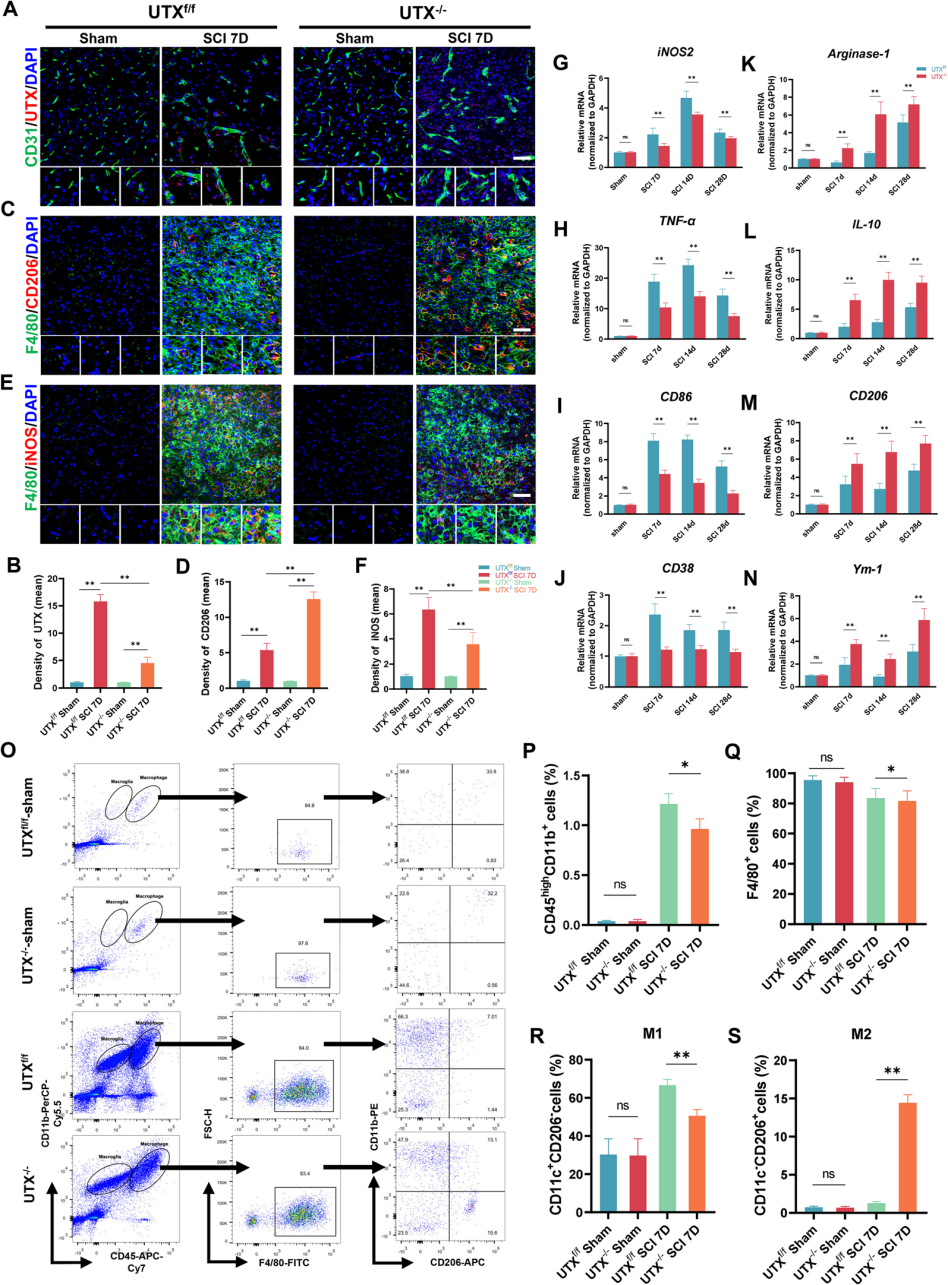
The UTX^−/−^ ECs promote macrophages polarization toward M2 subtype after SCI. **A** Immunofluorescence analysis of the UTX (red) expression in CD31^+^ endothelial cells (green) in sham, UTX^f/f^, and UTX^−/−^ mice at 7 days after SCI. Scale bar, 20 μm. **B** Statistical analysis of the mean fluorescence intensity of UTX in **A**. **C** Immunofluorescent co-staining of the M2 marker (CD206, red) and F4/80^+^ macrophages (green) in sham, UTX^f/f^ and UTX^−/−^ mice at 7 days post-SCI. Scale bars, 20 μm. **D** Statistical analysis of mean fluorescence intensity of CD206 in **C**. **E** Immunofluorescence analysis of the M1marker (iNOS, red) expression in F4/80^+^ macrophages (green) in sham, UTX^f/f^, and UTX^−/−^ mice at 7 days after SCI. Scale bars, 20 μm. **F** Statistical analysis of the mean fluorescence intensity of iNOS in **E**. **G**–**J** Relative mRNA expression of M1 macrophage markers (iNOS2, TNF-ɑ, CD86 and CD38). **K**–**N** Relative mRNA expression of M2 macrophage markers (Arg-1, IL-10, CD206 and Ym-1). **O** Representative scatter plots of macrophage phenotypes in sham, UTX^f/f^, and UTX^−/−^ mice at day 7 after SCI. Cells were immunolabeled with antibody-fluorophore coupled antibodies to CD45-APC-Cy7, CD11b-PerCP-Cy5.5, F4/80-FITC, CD206-APC, and CD11c-PE. Macrophages were defined as CD45^high^CD11b^+^, F4/80^+^ cells were defined as mature macrophages, and CD206 and CD11c defined macrophage phenotypes, M0 macrophages (CD11c^−^CD206^−^), M1 macrophages (CD11c^+^CD206^−^) and M2 macrophages (CD11c^−^CD206^+^). The numbers in the gates refer to the percentage of positive cells for each marker. **P** The percentage of macrophages (CD45^high^CD11b^+^) in **O**. **Q** The percentage of F4/80^+^ mature macrophage cells in the CD45^high^CD11b^+^ gate (macrophages). **R**, **S** The percentage of M1 macrophages (CD11c^+^CD206^−^) and M2 macrophages (CD11c^−^CD206^+^) cells in the CD45^high^CD11b^+^F4/80^+^ gate (mature macrophages). ^ns^*P* > 0.05, *P < 0.05, **P < 0.01, compared with corresponding control mice. n = 5/per group

To further explore the effect of UTX^−/−^ ECs on macrophages, we investigated the expression of UTX in ECs and the macrophage phenotypes in the injured region post-SCI. Firstly, the immunofluorescence results have shown that the level of UTX expression in CD31^+^ ECs of UTX^f/f^ mice was significantly increased at 7 days after SCI compared with Sham mice and UTX^−/−^ mice (Fig. 2A and B), which was consistent with the findings of our previous study [25]. Additionally, immunofluorescence was also performed to investigate the macrophage phenotypes. The results reveal that the number and mean fluorescence intensities of F4/80^+^CD206^+^ (M2) macrophages have significantly increased in UTX^−/−^ mice at 7 days post-SCI than that in UTX^−/−^ mice, while the iNOS^+^CD206^+^ (M1) macrophages were fewer (Fig. 2C–F). To further clarify whether the macrophage phenotypes changed in the injured epicenter at different phases following SCI in UTX^−/−^ mice, the qPCR was performed to detect the M2 and M1 macrophages’ markers. The results showed that the relative mRNA expression of M1-type markers (iNOS2, TNF-ɑ, CD86, and CD38) was decreased significantly in UTX^−/−^ mice following SCI and still higher than that of the UTX^f/f^ mice on day 28 post-SCI. The markers of M2-type (Arg-1, CD206, IL-10, and Ym-1) were precisely the opposite. These expressions were remarkedly higher in UTX^−/−^ mice after SCI compared to UTX^f/f^ mice (Fig. 2G–N). In addition, immunofluorescence and qRT-PCR were not clearly distinguished between the macrophages and microglia, but flow cytometry can be better distinguished by the level expression of CD45 and CD11b. The flow cytometry results revealed that the proportions of mature macrophages (CD45^high^CD11b^+^) were the same in the Sham group of UTX^−/−^ and UTX^f/f^ mice. These two group mice all significantly increased at 7 days post-SCI, but the number of macrophages in the injured region of UTX^−/−^ mice has slightly lower than that of UTX^f/f^ mice (Fig. 2O and P). Interestingly, the percentage of mature macrophages (CD45^high^CD11b^+^F4/80^+^) has no significant difference between these two groups (Fig. 2Q). These results indicated that UTX^−/−^ ECs might slightly influence the recruitment of macrophages but have no effect on macrophage maturation after SCI, which needed further research. Next, we assessed the proportions of M1 macrophages (CD11c^+^CD206^−^) and M2 macrophages (CD11c^−^CD206^+^) of UTX^−/−^ and UTX^f/f^ mice at 7 days post-SCI. The outcome has shown that the percentage of M1 macrophages (CD11c^+^CD206^−^) of UTX^−/−^ mice had remarkedly lower in the injured epicenter than that of UTX^f/f^ mice (66.7 ± 5.3% vs 47.9 ± 3.8%), while the proportion of M2 macrophages (CD11c^−^CD206^+^) was significantly higher compared to UTX^f/f^ mice (1.4 ± 0.72% vs 15.6 ± 3.4%) (Fig. 2 O, R, and S). These above findings suggest that the UTX^−/−^ ECs can promote macrophage polarization toward the M2 type after SCI.

### Exosomes mediated the EC-macrophage crosstalk

**Fig. 3:**
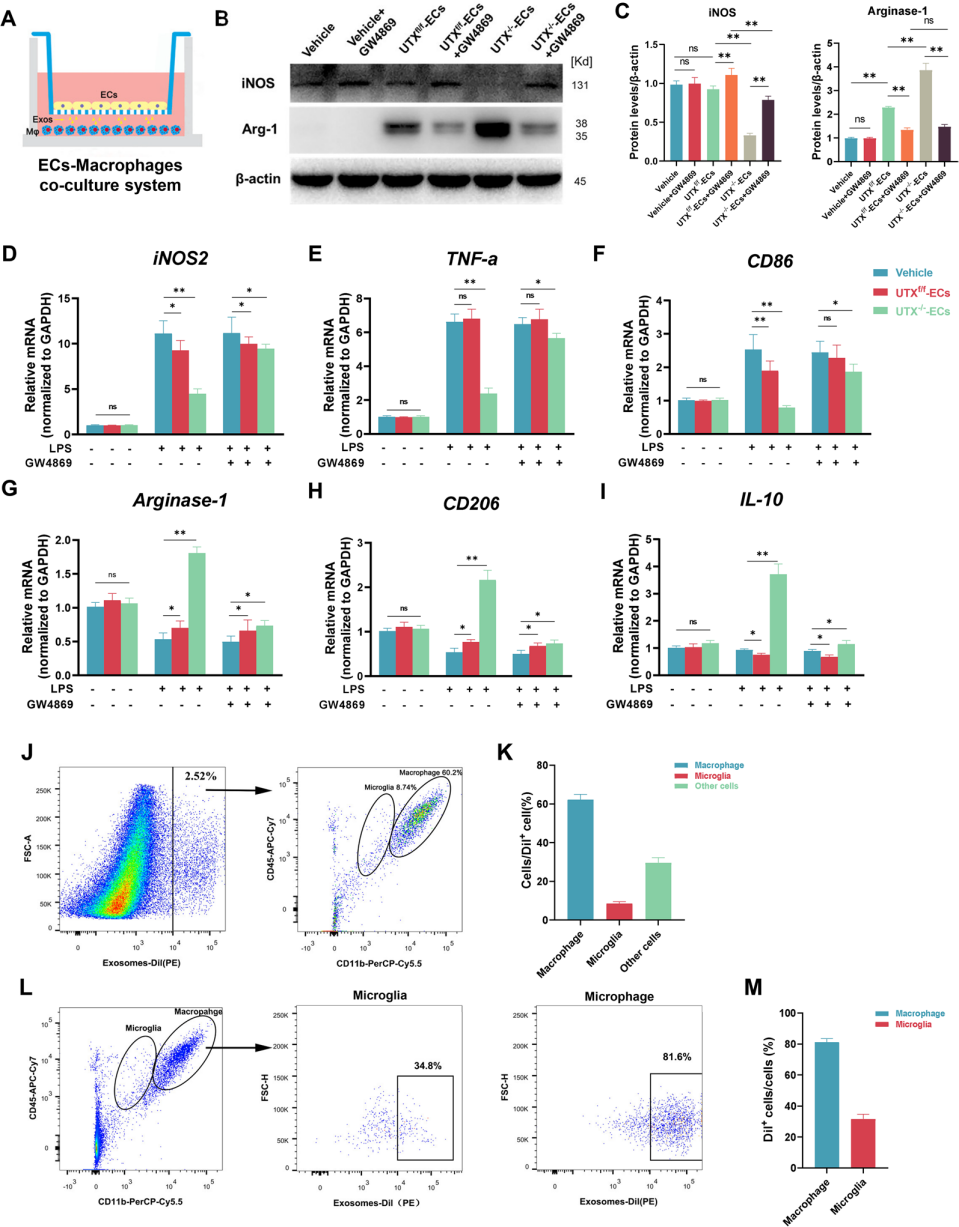
Exosomes mediated the EC-macrophage crosstalk. **A** Schematic diagram of the EC-macrophage co-culture system, upper layer: ECs; lower layer: macrophages. **B** Western blotting analysis of the expression of iNOS (M1 macrophage marker) and Arg-1 (M2 macrophage marker) in Vehicle, UTX^f/f^ ECs, and UTX^−/−^ ECs with the addition of GW4869. **C** Statistical analysis of iNOS and Arg-1 expression in each group in **B**. **D**–**I** qRT-PCR analysis of the mRNA expression of M1 markers (iNOS2, TNF-ɑ and CD86) and M2 markers (Arg-1, IL-10 and CD206) in Vehicle, UTX^f/f^ ECs and UTX^−/−^ ECs with addition of GW4869. **J** Representative scatter plot of WT mice taken up Dil-labeled exosomes on day 7 after SCI. Cells were immunolabelled with CD45-APC and CD11b-PerCP-Cy5.5 antibodies. Dil^+^ cells are defined as the cells taken up Dil-labelled ECs-Exos, macrophages defined as CD45^high^CD11b^+^, and microglial cells defined as CD45^low^CD11b^+^. The numbers in the gates refer to the percentage of positive cells for each marker. **K** Statistical analysis of the percentage of macrophages, microglia, and other cells taken-up Dil-labelled ECs-Exos in CD45 and CD11b gate. **L** Representative scatter plot of macrophage uptake of Dil-labeled ECs-Exos on day 7 post-SCI. The numbers in the gates refer to the percentage of positive cells for each marker. **M** Statistical analysis of Dil^+^ macrophages and Dil^+^ microglia percentage in Dil^+^ gate. ^ns^*P* > 0.05, *P < 0.05, **P < 0.01, compared with corresponding control mice. n = 5/per group

To further investigate how the UTX^−/−^ ECs promotes macrophage polarization, we isolated the primary endothelial cells and LPS-stimulated macrophages and then conducted an EC-macrophage co-culture system (Additional file 1: Fig. S2 A–F, Fig. 3A). Firstly, the western blotting result has shown that the expression of iNOS was remarkably lower in the UTX^−/−^ ECs group than in the UTX^f/f^ ECs group, whereas Arg-1’s expression was significantly higher. Furthermore, we confirmed the effect of GW4869, which can inhibit the release of cells’ exosomes, on inhibiting ECs-derived exosomes by western blotting. The result shows GW4869 can significantly inhibit the ECs from releasing exosomes (Additional file 1: Fig. S3 C). When the GW4869 was added to ECs’ medium, UTX^−/−^ ECs on promoting macrophages toward the M2 subtype were blocked (Fig. 3B and C). In addition, consistent with western blotting, the qRT-PCR results revealed that there have no statistically significant differences in the mRNA expressions of M1 (iNOS2, TNF-ɑ, CD86) or M2 (Arg-1, CD206, IL-10) markers between the three groups of Vehicle, UTX^f/f^ ECs, and UTX^−/−^ ECs when the macrophages were not stimulated with LPS. However, after LPS stimulation, the mRNA expressions of M1-type markers were significantly decreased, and the M2 markers were remarkedly increased in the UTX^−/−^ ECs group compared to the Vehicle and UTX^f/f^ ECs group. Following the addition of GW4869 in the ECs medium, the decreasing trend of M1-type markers and the increasing trend of M2 markers in the UTX^−/−^ ECs group were blocked (Fig. 3D–I). Interestingly, we found the ECs also slightly affect macrophage polarization, consistent with previous studies [13]. According to these results, the UTX^−/−^ ECs can mediate EC-macrophage crosstalk by exosomes and promotes LPS-stimulated macrophages to the M2 subtype.

### Macrophages were the major cells that take-up ECs-derived exosomes

To explore whether macrophages take up ECs-derived exosomes (ECs-Exos), we first isolated and characterized the exosomes from ECs. The TEM, western blotting (TSG101, CD63, and Calnexin as exosome markers), and NTA results have been shown (Additional file 1: Fig. S3A–C). Furthermore, we determined whether macrophages could take up ECs-Exos. As shown in Additional file 1: Fig. S3D, Dil-labelled ECs-Exos were internalized into the perinuclear region of macrophages after 4 h incubation. Additionally, we used a laser scanning confocal fluorescence microscope for further analysis to eliminate the bias of the experimental results caused by residual Dil reagent staining the cell membrane. The result further confirmed that Dil-labelled ECs-Exos appears as a "cloud" around the nucleus of macrophages (Additional file 1: Fig. S3E). In vivo tracer experiments revealed a significant overlap in the distribution areas of F4/80^+^ macrophages and Dil-labeled ECs-Exos in the injured epicenter after SCI (Additional file 1: Fig. S3F). These findings suggested that macrophages could take up ECs-derived exosomes.

To further explore whether macrophages were the major cells that take up ECs-Exos in vivo, we performed flow cytometry to detect the percentage of macrophages taking up Dil-labeled ECs-Exos. Firstly, we discovered that the cells taken up Dil-labeled ECs-Exos accounted for 2.57 ± 0.26% of the total number of cells at days 7 post-SCI. Macrophages (CD45^high^CD11b^+^) made up 60.2 ± 5.3% of them, while microglia (CD45^low^CD11b^+^) made up only 8.74 ± 1.05% (Fig. 3J, K). After reanalyzing the flow cytometry results, we separated macrophages and microglia by the CD45 and CD11b gate. Figures 3L and M have shown that Dil^+^ microglia accounted for 35.2% of the total microglia, while Dil^+^ macrophages accounted for 81.54% of the total macrophages in the injured region. These findings indicate that macrophages may be the major cells that uptake exosomes produced by ECs in the injured site following SCI.

### In vitro and in vivo, UTX^−/−^-Exos promote macrophage polarization and improve neurological recovery post-SCI

**Fig. 4:**
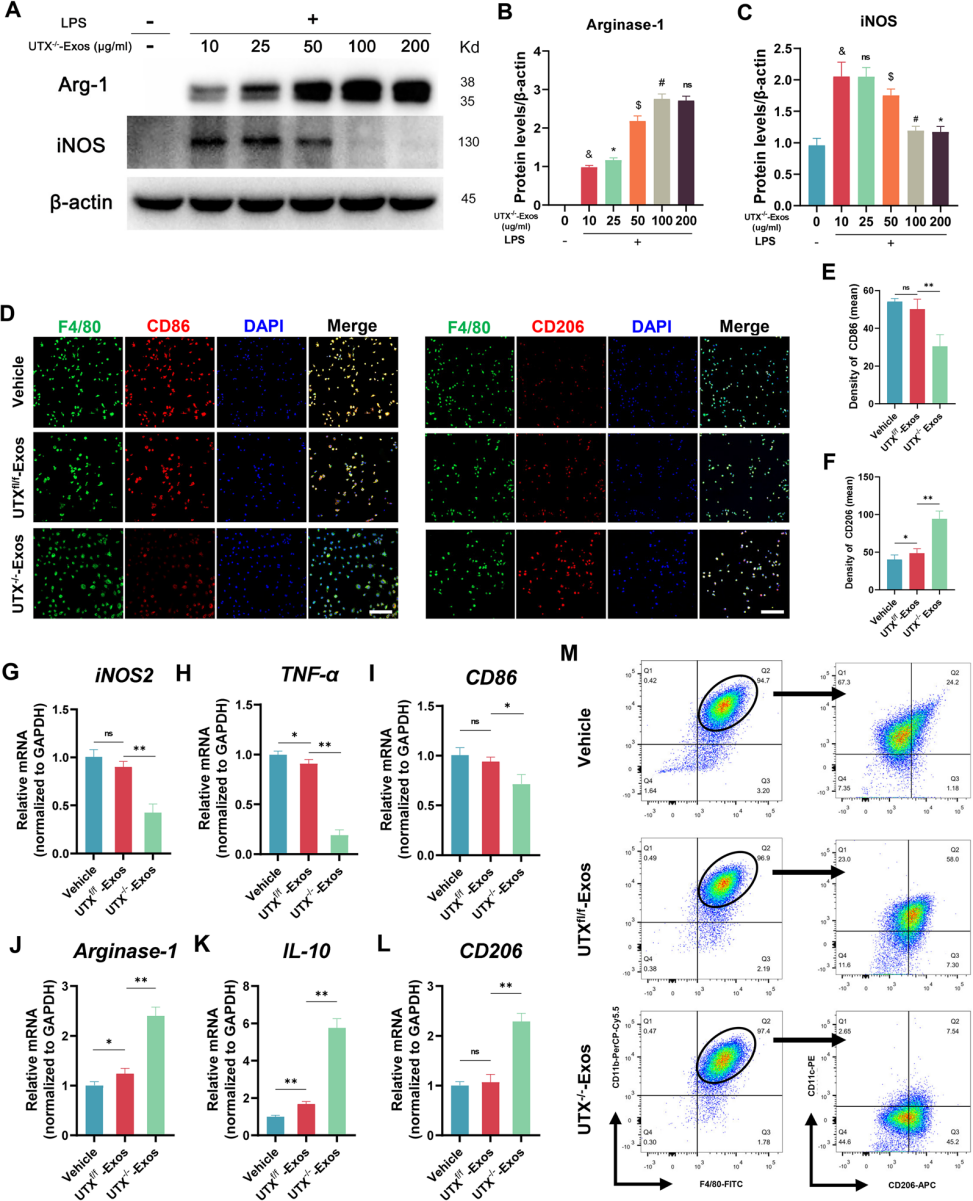
UTX^−/−^ ECs-Exos promotes macrophage polarization in vitro. **A** Western blotting analysis of the iNOS (M1 marker) and Arg-1 (M2 marker) expression of LPS-stimulated macrophages after being treated with different concentrations of UTX^−/−^-Exos. **B** Statistical analysis of the Arg-1 expression in **A**. ^&^*P* < 0.05 vs*.* 0 μg/ml UTX^−/−^-Exos, *P < 0.05 vs. 10 μg/ml UTX^−/−^-Exos, ^$^*P* < 0.05 vs. 25 μg/ml UTX^−/−^-Exos, ^#^*P* < 0.05 vs. 50 μg/ml UTX^−/−^-Exos, ^ns^*P* > 0.05 vs*.* 100 μg /ml UTX^−/−^-Exos. **C** Statistical analysis of iNOS expression in **A**. **D** Immunofluorescent co-staining of the CD86 (M1)/CD206 (M2) (red) and F4/80^+^ macrophages (green) after being treated with Vehicle, UTX^f/f^-Exos, and UTX^−/−^-Exos. Scale bars, 20 μm. **E** Statistical analysis of the mean fluorescence intensity of CD86 in **D**. **F** Statistical analysis of the mean fluorescence intensity of CD206 in **D**. **G**–**L** qRT-PCR analysis of the mRNA expression of M1 markers (iNOS2, TNF-ɑ, and CD86) and M2 markers (Arginase-1, IL-10, and CD206) of LPS-stimulated macrophages after treated with Vehicle, UTX^f/f^-Exos, and UTX^−/−^-Exos. **M** Representative scatter plots of the percentage of macrophage phenotypes when exposed to the Vehicle, UTX^f/f^-Exos, and UTX^−/−^-Exos. Mature macrophages are defined as F4/80^+^CD11b^+^, M1 macrophages are defined as CD11c^+^CD206^−^, and M2 macrophages are defined as CD11c^−^CD206^+^. The numbers in the gates refer to the percentage of positive cells for each marker. ^ns^*P* > 0.05, *P < 0.05, **P < 0.01, compared with corresponding control mice. n = 6/per group

To determine whether UTX^−/−^ ECs-derived exosomes (UTX^−/−^-Exos) could promote macrophage polarization toward the M2 subtype, we first determine the optimal effective exosome concentration by western blotting. The results showed that the effect of UTX^−/−^-Exos increased with the concentration, and at 100 mg/ml and 200 mg/ml, the expression of iNOS and Arg-1 was not statistically significant (Fig. 4A–C). Thus, 100 g/ml might be the most suitable dose for further investigation. Furthermore, immunofluorescence (Fig. 4D–F), qRT-PCR (Fig. 4G–L), and flow cytometry (Fig. 4 M) was also performed*.* In conclusion, these results confirmed that UTX^−/−^-Exos enhances M2 macrophage polarization in vitro while lowering M1 macrophage polarization.

**Fig. 5:**
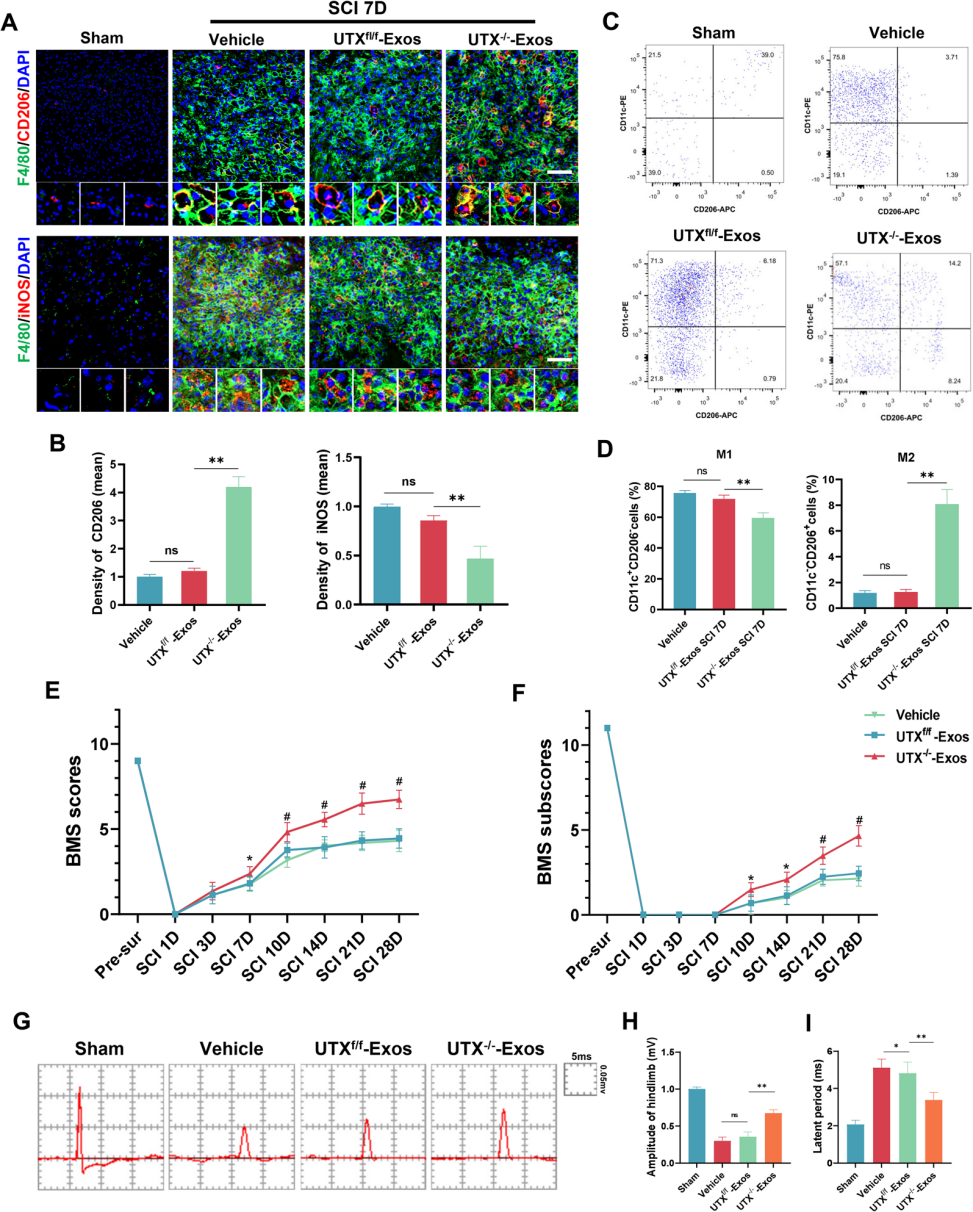
UTX^−/−^ ECs-Exos promotes macrophage polarization and neurological recovery after SCI. **A** Immunofluorescence analysis of the level expression of CD206^+^ (M2)/iNOS^+^ (M1) (red) in F4/80^+^ macrophages (green) in the Sham, Vehicle, UTX^f/f^-Exos, and UTX^−/−^-Exos groups at 7 days after SCI. Scale bar, 20 μm. **B** Statistical analysis of mean fluorescence intensity of CD206 and iNOS in **A**. **C** Representative scatter plots of macrophage phenotypes in sham, Vehicle, UTX^f/f^-Exos, and UTX^−/−^-Exos groups at 7 days after SCI. Cells were immunolabeled with antibody-fluorophore-coupled antibodies for CD206 and CD11c. M1 macrophages are defined as CD11c^+^CD206^−^ and M2 macrophages are defined as CD11c^−^CD206^+^. The numbers in the gates refer to the percentage of positive cells for each marker. **D** The percentage of M1 macrophages (CD11c^+^CD206^−^) and M2 macrophages (CD11c^−^CD206^+^) cells in **C**. **E**, **F** Distribution of the BMS scores and subscores per group at Pre-sur, 1, 3, 7, 10, 14, 21, and 28 days post-SCI. n = 8/per group. **G** Representative electrophysiological traces in each group at 28 days post-SCI. **H, I** Quantification of MEP amplitude and latent period in **G**. ^ns^*P* > 0.05, *P < 0.05, **P < 0.01 vs*.* corresponding control mice. n = 5/per group

We further determined the effects of UTX^−/−^-Exos on regulating macrophage polarization in vivo by immunofluorescent staining assays and flow cytometry*.* The immunofluorescent staining demonstrated an increased level of M2 macrophages (F4/80^+^CD206^+^) and a decrease in M1 macrophages (iNOS^+^CD206^+^) in the UTX^−/−^ ECs-Exos group after SCI as compared to the Vehicle and UTX^f/f^-Exos group (Fig. 5A, B). In addition, consistent with the immunofluorescence result, the flow cytometry further confirmed that compared to the SCI mice treated with UTX^−/−^-Exos, the percentage of M1 macrophages (CD11c^+^CD206^−^) was remarkedly lower (73.3 ± 4.5% vs*.* 57.4 ± 2.9%) after treated with UTX^−/−^-Exos at 7 days post-SCI, while M2 macrophages (CD11c^−^CD206^+^) was significantly higher (1.03 ± 0.45% vs*.* 8.4 ± 1.2%) (Fig. 5C, D). These results support the hypothesis that exosomes derived from UTX^−/−^ ECs may influence macrophage polarization towards M2. Behavior testing was conducted to evaluate the effects of UTX^−/−^-Exos on tissue repair and functional recovery after SCI. As shown in Fig. 5E–I, UTX^−/−^-Exos benefited the recovery of motor function and neurophysiological activity in the hindlimbs after SCI.

### UTX^−/−^ ECs may not impact the microglia polarization

Besides macrophages, microglia, innate immune cells in the spinal cord, are present in the injured epicenter after SCI. The microglia would also shift their phenotype in response to environmental changes. Although immunofluorescence and qRT-PCR are insufficient to distinguish between macrophages and microglia, flow cytometry can distinguish macrophages (CD45^high^ CD11b^+^) from microglia (CD45^low^ CD11b^+^) based on their levels of CD45 expression. The results have shown that there was no statistically significant difference between UTX^f/f^ mice and UTX^−/−^ mice concerning the percentages of microglia (CD45^low^CD11b^+^), activated microglia (F4/80^−^), M1 microglia (CD11c^+^CD206^−^), and M2 microglia (CD11c^−^CD206^+^). Additionally, as shown in Additional file 1: Fig. S4A, although the number and percentage of microglia sharp rise, the percentages of microglia, activated microglia, M1 and M2 microglia have no statistically significant difference between the two groups in the injured region on day 7 after SCI (Additional file 1: Fig. S4A–E). To determine whether UTX^−/−^-Exos affect the microglia polarization, we performed qRT-PCR to detect the M1 and M2 markers of LPS-stimulated microglia after being treated with UTX^−/−^-Exos. Interestingly, we found UTX^−/−^-Exos can promote microglia toward its M2 subtype (Additional file 1: Fig. S4 F–K). A possible explanation for the different effects between in vivo and in vitro may be that macrophages mainly took up the exosomes derived from UTX-ECs in the epicenter of the injury after SCI. However, further research is necessary to determine the exact mechanism.

### Exosomal miR-467b-3p levels are associated with macrophage polarization

**Fig. 6:**
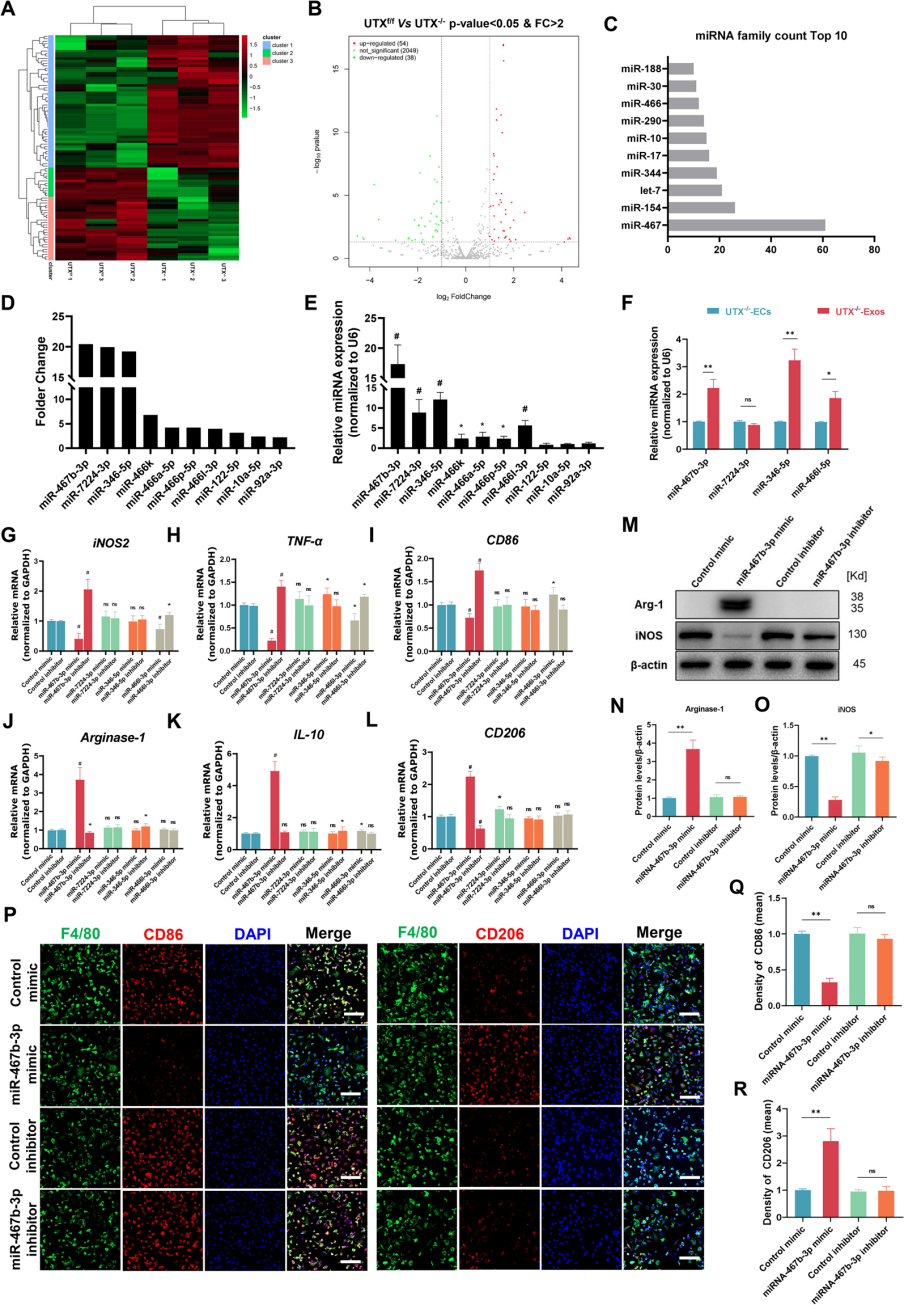
Exosomal miR-467b-3p levels are associated with macrophage polarization. **A** Hierarchical cluster heatmap of the differential miRNAs in UTX^−/−^-ECs compared to UTX^f/f^-ECs. Each cell represents a differentially expressed miRNA. The color scale from green (low) to red (high) indicates the expression levels of DEPs. **B** Volcano plot of differentially expressed miRNA (*P* value < 0.05 and log_2_ Fold-Change > 1) UTX^−/−^-ECs compared with UTX^f/f^-ECs. **C** The Top10 miRNAs family of differentially expressed miRNAs. **D** The expression levels top10 up-regulated candidate miRNAs. **E** The miRNAs’ expression level in UTX^−/−^-Exos verified by qRT-PCR. *P < 0.05, ^#^*P* < 0.01 vs*.* UTX^f/f^-Exos. **F** The enrichment of miRNAs in UTX^−/−^-Exos verified by qRT-PCR. ^ns^*P*>0.05, *P<0.05, **P<0.01 vs. UTX^−/−^-ECs. **G–L** qRT-PCR analysis of the expression of M1 markers (iNOS2, TNF-ɑ, and CD86) and M2 markers (Arginase-1, IL-10, and CD206) of LPS-stimulated macrophages after being treated with the mimics and inhibitors of the miRNAs (miR-467b-3p, miR-7224-3p, miR-346-5p, and miR-466l-3p). **M** The iNOS and Arginase-1 expression of LPS-stimulated macrophages when exposed to the mimic and inhibitor of miR-467b-3p compared to the control mimic/inhibitor. **N, O** Statistical analysis of Arginase-1 and iNOS expression in **M**. **P** Immunofluorescent analysis of LPS-stimulated macrophage (green) phenotypes (red) when exposed to the mimic and inhibitor of miR-467b-3p compared with control mimic and inhibitor. Scale bar, 20 μm. **Q, R** Statistical analysis of mean fluorescence intensity of CD86 and CD206 in **P**. ^ns^*P* > 0.05, *P < 0.05, **P < 0.01 vs*.* Control mimic/inhibitor. n = 6/per group

We performed miRNA sequencing to detect miRNA expression to explore further the functional molecule of UTX^−/−^-Exos regulating macrophage polarization. As the hierarchical cluster heatmap and volcano plot revealed in Fig. 6A, B, 92 differentially expressed miRNAs were identified. Among them, 54 miRNAs in UTX^−/−^ ECs were significantly higher, and 38 miRNAs were much lower than in UTX^f/f^ ECs. Furthermore, the known miRNA family’s information was analyzed by miRBase data, and the miR-467 family was the most abundant (Fig. 6C). We selected a total of 10 miRNAs for further analysis, including the top six most significantly up-regulated miRNAs (miR-467b-3p, miR-7224-3p, miR-346-5p, miR-466k, miR-466a-5p,miR-466p-5p), three associated with macrophage polarization (miR-122-5p, miR-10a-5p, and miR-92a-3p), and one related to the release of inflammatory factor from macrophages (miR-466l-3p) (Fig. 6D). In addition, qRT-PCR also confirmed the enrichment of four miRNAs (miR-467b-3p, miR-7224-3p, miR-346-5p, miR-466l-3p) in UTX^−/−^-Exos (Fig. 6E and F).

Next, we constructed the four enrichment miRNAs' mimics and inhibitors to reveal which miRNAs in UTX^−/−^ ECs-Exos mediated macrophage polarization. And then, we analyzed their effects on macrophage polarization by qRT-PCR. Based on the findings of qRT-PCR, miR-467b-3p increased the markers of M2 macrophages while inhibiting those of M1 macrophages (Fig. 6G–L). Furthermore, western blotting and immunofluorescent staining results also confirmed that miR-467b-3p inhibits macrophage polarization toward the M1 subtype and promotes toward the M2 subtype (Fig. 6M–R). The above outcomes suggest that the exosomal miR-467b-3p from UTX^−/−^ ECs are associated with macrophage polarization.

### UTX directly regulate miR-467b-3p and inhibit the expression of PTEN

**Fig. 7:**
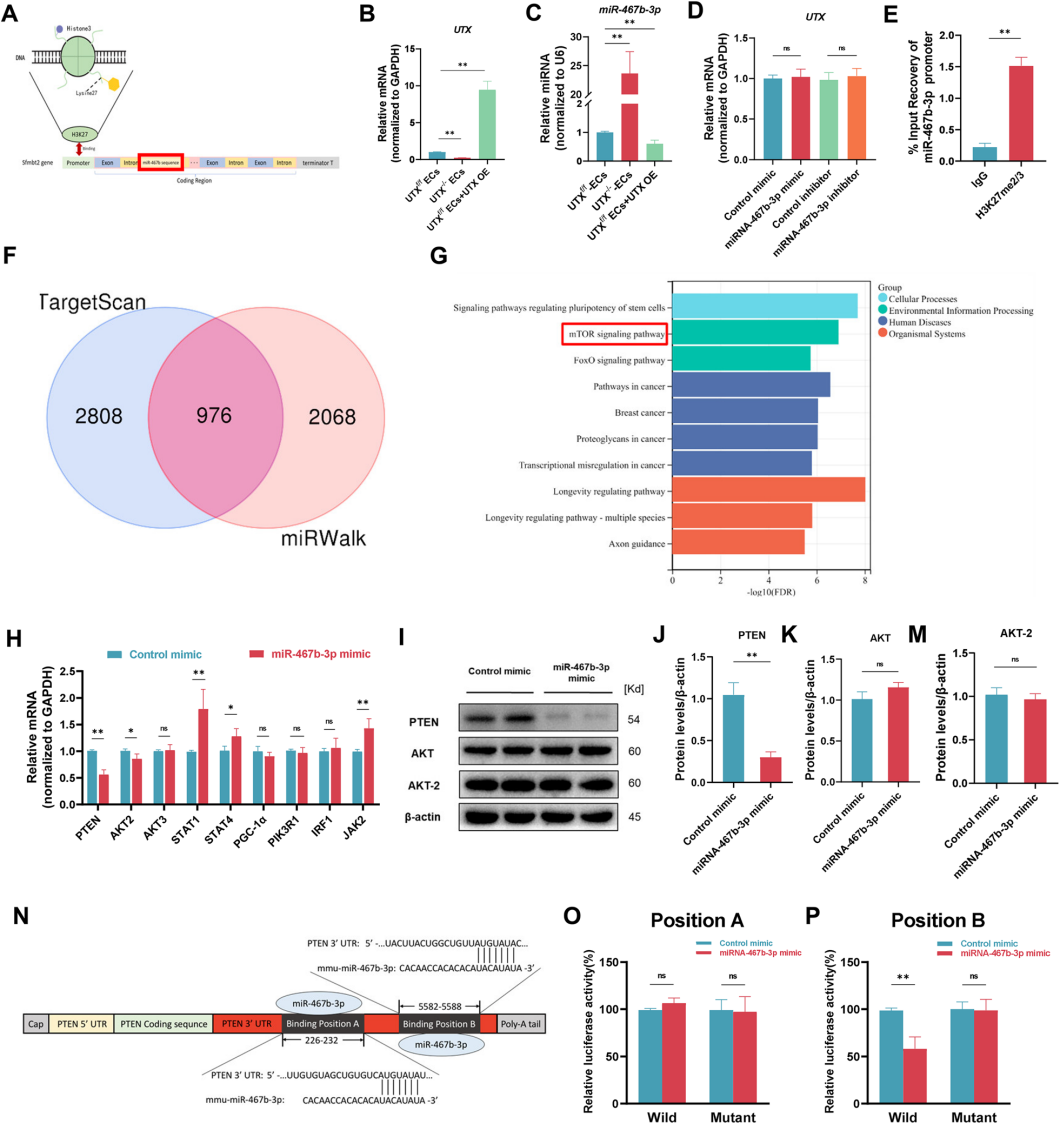
UTX directly regulate miR-467b-3p and inhibit the expression of PTEN. **A** A schematic illustration of the UTX, H3K27me3/me2 and miR-467b-3p/Smfbt2 promoter forming an epigenetic regulation complex. **B** The overexpression efficiency of UTX verified by qRT-PCR. **C** The expression levels of miR-467b-3p in UTX^f/f^ ECs, UTX^−/−^ ECs and UTX^f/f^-ECs + UTX OE. **D** The mRNA expression levels of UTX after miR-467b-3p mimic and inhibitor conducted to ECs. **E** Chip-qPCR detection of the rate of H3K27 binding to miR-467b-3p/Sfmbt2 promotor. **F** Overlap analysis of predicted potential target proteins of miR-467b-3p from the TargetScan database and miRWalk database. **G** KEGG signaling pathway enrichment analysis. **H** qRT-PCR analysis of the mRNA expression levels of candidate target proteins after miR-467b-3p mimic transfected macrophages. **I** Western blotting analysis of the levels of PTEN, AKT and AKT2 after miR-467b-3p mimic transfected macrophages. **J**–**M** Statistical analysis of PTEN, AKT and AKT2 expression levels in **I**. **N** Schematic diagram of the binding site of miR-467b-3p to PTEN mRNA. **O**, **P** Dual luciferase reporter assay to detect luciferase activity in wild-type (Wild) and mutant (Mutant) PTEN loci after miR-467b-3p mimic transfection of HEK-293T cells. ^ns^*P* > 0.05, **P < 0.01 vs*.* corresponding control groups. n = 6/per group

Experimental results suggest a close regulatory relationship between UTX and miR-467b-3p in ECs. To further clarify this relationship, we first obtained the bioinformatics of miR-467b-3p from the UCSC database and found it was located within the Sfmbt2 intron. The latest study shows that the demethylation of histone H3K27me3 can promote the expression of the miRNA cluster of Sfmbt2 [23]. Therefore, we speculate that UTX, H3K27me3, and Sfmbt2 form an epigenetic regulatory complex (Fig. 7A). Next, we up-regulated the expression of UTX in UTX^f/f^ ECs by transfecting the UTX overexpressing lentivirus and confirmed by qRT-PCR analysis (Fig. 7B). Moreover, we performed western blotting to further verify whether UTX-KO influenced the level of H3K27me3/me2 in ECs. The result of western blotting showed that compared to UTX^f/f^ ECs, the expression level of H3K27me3/me2 was significantly higher in UTX^−/−^ ECs (Additional file 1: Fig. S6 A and B), indicating the level of H3K27me3/me2 were increased after UTX-KO in ECs. As shown in Fig. 7C, miR-467b-3p expression was directly correlated with the level of UTX expression. However, the up-and down-regulation of miR-467b-3p does not affect the mRNA expression of UTX (Fig. 7D). Furthermore, to further verify the regulatory relationship between UTX and miR-467b-3p in the SCI mice model, we performed qRT-PCR to detect the expression level of miR-467b-3p following SCI. These results have shown that miR-467b-3p expression levels in the injured region of the spinal cord were significantly decreased at 7 days post-SCI compared to the UTX^f/f^ sham mice. However, miR-467b-3p levels were significantly higher in the injured epicenter of the spinal cord 7 days after SCI in the UTX^−/−^ mice compared to the UTX^f/f^ mice, and miR-467b-3p expression was even lower than UTX^f/f^ mice after local transduce with UTX overexpressing lentivirus in UTX^−/−^ mice, indicating that UTX knockdown in Tek^+^ ECs also can regulate the miR-467b-3p expression level in the injured site of the spinal cord after SCI (Additional file 1: Fig. S6 C). In addition, we performed the Chip-qPCR to determine the relationship between H3K27 and the Sfmbt2/miR-467b-3p promoter. This result demonstrated a direct binding interaction between H3K27me3 and the Sfmbt2/miR-467b-3p promoters (Fig. 7E). In conclusion, these outcomes suggested that UTX directly regulates miR-467b-3p expression by forming an epigenetic regulatory complex.

To reveal the downstream mechanism of the miR-467b-3p mediated macrophage polarization, we first predicted the target of miR-467b-3p by the TargetScan and miRWalk databases (Fig. 7F) and then performed GO and KEGG enrichment analysis of these overlapped data. The KEGG analysis suggested that these possible targets of miR-467b-3p are tightly related to the mTOR signaling pathway, which was associated with macrophage polarization (red box) (Fig. 7G). Interestingly, GO enrichment analyses found that most of the predicted targets of miR-467b-3p concerned the mTOR signaling pathway (Additional file 1: Fig. S5 A and B). Therefore, the bioinformatics analysis indicates that the possible target of miR-467b-3p may influence macrophage polarization via the mTOR signaling pathway. Following these analyses, we selected nine potential targets for miR-467b-3p (PTEN, AKT2, AKT3, STAT1, STAT4, PGC-1, PIK3R1, IRF1, and JAK2), all of which were involved in the mTOR signaling pathway. The results of qRT-PCR and western blotting revealed treatment with miR-467b-3p mimic could decrease the levels of PTEN, indicating the potential target of miR-467b-3p regulating macrophage polarization might be PTEN, a negative regulatory factor of the mTOR signaling pathway (Fig. 7H–M). Moreover, to further verify whether UTX^−/−^-Exos regulates the PTEN expression in macrophages, we performed western blotting and qRT-PCR to detect the level of PTEN after UTX^−/−^-Exos treating LPS-stimulated macrophages. These results have shown that PTEN expression levels significantly decreased in LPS-stimulated macrophages after UTX^−/−^-Exos treatment compared with the UTX^f/f^-Exos group. In contrast, the decrease of PTEN expression was blocked in the UTX^−/−^-Exos + UTX OE group, indicating the exosomes derived from UTX deletion ECs can also decrease the PTEN expression level in LPS-stimulated macrophages (Additional file 1: Fig. S6 D−F). Based on the Targetscan database, we predict that miR-467b-3p may be directly bound to two sites in the PTEN 3' UTR, located in the highly conserved regions 226–232 (Position A) and 5582–5588 (Position B), respectively (Fig. 7N). Dual luciferase reporter assay showed that the activity of luciferase reporters of position B was markedly reduced by miR-467b-3p overexpression as compared with controls; in contrast, the luciferase reporter of position A was not affected by the miR-467b-3p mimic, demonstrating that mi-467b-3p could directly interact with 30-UTR of PTEN (Fig. 7O, P).

### UTX/miR-467b-3p activates the PTEN/PI3K/mTOR pathway to mediate macrophage polarization

**Fig. 8:**
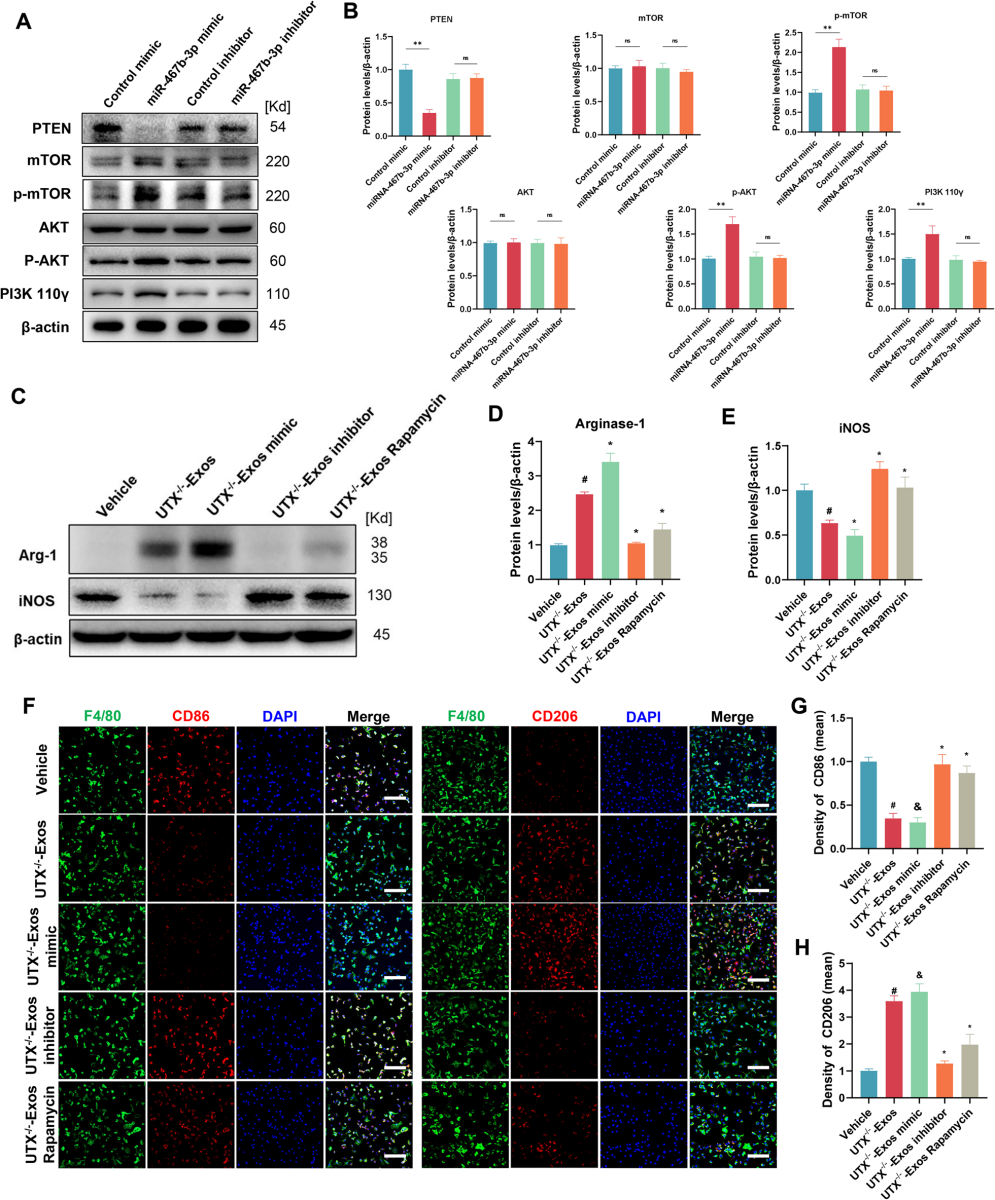
UTX/miR-467b-3p activates the PTEN/PI3K/mTOR pathway to mediate macrophage polarization. **A** Western blotting analysis of the expression levels of PTEN/PI3K/ mTOR signaling pathway-related proteins after miR-467b-3p mimic and inhibitor transfection of LPS-stimulated macrophages. **B** Statistical analysis of the expression levels of the PTEN/PI3K/mTOR signaling pathway-related proteins in **A**. **C** Western blotting analysis of the expression levels of iNOS and Arginase-1 of LPS-stimulated macrophages in Vehicle, UTX^−/−^-Exos, UTX^−/−^-Exos mimic, UTX^−/−^-Exos inhibitor, and UTX^−/−^-Exos with rapamycin groups. **D**, **E** Statistical analysis of iNOS and Arginase-1 expression levels in **C**. ^#^*P* < 0.01 vs. Vehicle group, *P < 0.01 vs. UTX^−/−^-Exos group. **F** Immunofluorescence analysis of LPS-stimulated macrophages (green) phenotypes (red) in Vehicle, UTX^−/−^-Exos, UTX^−/−^-Exos mimic, UTX^−/−^-Exos inhibitor, and UTX^−/−^-Exos with rapamycin groups. Scale bar, 20 μm. **G**, **H** Statistical analysis of the mean fluorescence intensity of CD86 and CD206 in **F**. ^#^*P* < 0.01 vs. Vehicle group, ^&^*P* < 0.05 vs*.* UTX^−/−^-Exos group, *P < 0.01 vs*.* UTX^−/−^-Exos group

To further elaborate whether UTX/miR-467b-3p activates PTEN/PI3K/mTOR signaling pathway to regulate macrophage polarization, we first detect the expression levels of key proteins of the PTEN/PI3K/mTOR signaling pathway after miR-467b-3p mimic and inhibitor transfection of macrophages. The results showed that miR-467b-3p phosphorylated and activated the PI3K/mTOR signaling pathway by targeting the inhibition of PTEN expression (Fig. 8A, B). To clarify whether UTX/miR-467b-3p activates the mTOR pathway and mediates macrophage polarization via exosomes, we transfected UTX^−/−^ ECs with mimic and inhibitor of miR-467b-3p and isolated their exosomes for further assay. The results of immunofluorescent staining and western blotting revealed that the expression of M2 markers (CD206 and Arg-1) was significantly increased. In contrast, the M1 markers (iNOS and CD86) decreased after being treated with UTX^−/−^-Exos and UTX^−/−^-Exos + miR-467b-3p mimic. And the results were reversed after adding miR-467b-3p inhibitor or rapamycin, which can inhibit the activity of the mTOR signaling pathway (Fig. 8C–H). These findings above show that UTX/miR-467b-3p regulates the PTEN/PI3K/mTOR signaling pathway to inhibit macrophage polarization toward the M1 subtype and enhance toward the M2 subtype.

## Discussion

SCI is a severe injury to the central nervous system that can set off several pathophysiological procedures and molecular events [2]. Epigenetic regulation, is a controlling gene transcription, plays a crucial role in the mammalian nervous system in response to trauma [30]. However, far too few studies have focused on SCI. Furthermore, previous studies are predominantly neurocentrically focused and considered insufficient regarding vascular immunity [31, 32]. Based on our previous studies, targeting vascular regeneration was a promising strategy for promoting functional recovery after SCI [25, 33, 34]. UTX deletion in Tek^+^ ECs can epigenetically promote vascular regeneration and functional recovery post-SCI [25].

Researchers have demonstrated that ECs cooperate with immune cells to produce cytokines, which are crucial to controlling immune responses by stimulating or inhibiting immune cells [12]. Additionally, ECs can crosstalk with macrophages to regulate neuroimmunity in the central nervous system [35–37]. In this study, we discovered that the promotion of neurological function in UTX^−/−^ ECs mice was significantly blocked on day 28 after SCI in a macrophage depletion model. This finding suggests that macrophages in the injured region may be crucial in improving neurological recovery in UTX^−/−^ ECs mice. Furthermore, recently, several studies have found that UTX can promote M2 macrophage polarization by directly activating classic cytokines, such as IL-6, Arg-1, IL-10, and Ym-1, in h3k27me3 dependent manner [38–43]. UTX also can increase the sensitivity of macrophages to interleukin 4-mediated polarization toward the M2 subtype [38]. In our study, consistent with the findings of these previous studies, M1 macrophage markers considerably decreased in the injured epicenter at various intervals post-SCI were observed in the UTX^−/−^ ECs mice, whereas M2 subtype markers were increased compared with the UTX^f/f^ ECs mice, indicating that UTX deletion in Tek^+^ ECs can regulate classic cytokines expressions in macrophages after SCI. Moreover, the flow cytometry analysis suggested that UTX^−/−^ ECs might also slightly influence the recruitment of macrophages but have no effect on macrophage maturation after SCI. These results indicated that UTX knockdown in Tek^+^ ECs promotes neurological recovery after SCI primarily by polarizing macrophages toward M2, while having little effect on macrophage recruitment and maturation. Thus, the intervention of UTX-mediated downstream signaling in ECs would have greater epigenetic significance for treating macrophage-related neurological dysfunction after SCI. However, its precise mechanism remains unclear.

As a key mediator of cell-to-cell communication, exosomes contain proteins and non-coding RNAs and regulate the biological processes taken up by recipient cells [16, 44–50]. Recently a study has reported that ECs can affect macrophage polarization via secreting exosomes [51]. In our study, we demonstrated that UTX deletion in ECs could regulate the polarization phenotype of macrophages in the "non-touch" EC-macrophage co-culture system. Notably, the experiment of exosome inhibition further confirmed that the exosomes derived from ECs might mediate the EC-macrophage crosstalk. Additionally, exosome uptake experiments revealed that macrophages are probably the main cells taking up EC-derived exosomes in the injured epicenter post-SCI, whereas microglia account for a small proportion. Then, we isolated UTX^−/−^ ECs-derived exosomes and further confirmed its function of promoting macrophage polarization toward the M2 subtype and reducing polarization toward the M1 subtype. Importantly, the UTX^−/−^ EC-Exos also promote the improvement of sensory and locomotive function post-SCI.

Aside from macrophages, microglia, innate immune cells in neural tissue, are also present in the injured region after SCI, both essential for repairing nervous damage [52, 53]. A microglia's polarization phenotype can also change based on the environment, exhibiting comparable biological activities [6]. In this study, UTX^−/−^ ECs primarily regulate macrophage polarization but not microglia in vivo. Interestingly, UTX^−/−^-Exos can promote microglia polarizes to the M2 subtype in vitro. There is a possibility that UTX^−/−^ ECs Exos could regulate microglia polarization; however, macrophages are the main recipients of exosomes derived from ECs in the injured epicenter after SCI, but further research is needed to clarify the exact mechanism.

An epigenetic modification plays a crucial role in regulating cell fate, maintaining cell specificity, and regulating cell type-specific functions during development and physiological changes. H3K27 methylation in gene promoters is a sign of repression, while H3K27me3 methylation is a sign of activation. UTX/KDM6A removes methyl groups from H3K27me3/me2, initiating transcription. According to recent studies, histone H3K27me3/me2 methylation regulates epigenetic functions by controlling the expression of several miRNA clusters [23, 54–56]. Therefore, we performed miRNA sequencing analysis to investigate the functional miRNA that mediated the effects of UTX^−/−^-Exos. Interestingly, compared with other macrophage polarization-associated miRNAs, we found significant enrichment of miR-467b-3p in UTX^−/−^-Exos. However, the role of UTX in regulating the miR-467b-3p expression in ECs has not been elucidated. This study found that H3K27me3/me2 demethylated by UTX may directly bind to miR-467b-3p/Sfmbt2 promoters, increasing miR-467b-3p expression. In vitro studies show that up-and down-regulation of miR-467b-3p affects macrophage biology and EC-macrophage crosstalk. Given UTX has been overexpressed in ECs after SCI, inhibition of miR-467b-3p, the downstream member of UTX signaling, is a promising therapeutic strategy for regulating the balance of macrophage immunophenotyping.

The principal mechanism of miRNAs inhibiting target protein is the 3' UTR of miRNAs binding to the mRNA of the downstream target protein and forming an RNA-induced silencing complex (RISC), preventing mRNA from translating [57, 58]. In this study, miR467b-3p is mainly expressed in the cytoplasm of macrophages, and its 3′ UTR could bind to the PTEN mRNA, providing the possibility that PTEN acts as the miR-467b-3p targeting protein. In vitro studies confirmed that intervention of miR467b-3p could affect the expression of PTEN. Recently, Lei et al. have reported that UTX has up-regulated the PTEN expression level, and UTX knockdown decreased the expression of PTEN, which promotes the proliferation and differentiation of neural stem cells by activating the PTEN-AKT-mTOR pathway [59]. Qi et al. have also found that UTX can enhance PTEN expression to decrease p-AKT and p-mTOR levels, promoting iPSC reprogramming efficiency by accelerating changes in the gene expression profile and the metabolic pattern in a demethylation-activity-dependent manner [60]. In this study, we found that PTEN expression levels significantly decreased in LPS-stimulated macrophages after UTX^−/−^-Exos treatment compared with the UTX^f/f^-Exos group. In contrast, the level of PTEN was higher than that in the UTX^f/f^-Exos + UTX OE group, indicating the exosomes derived from UTX deletion ECs can also decrease the PTEN expression level in LPS-stimulated macrophages.

PTEN (phosphatase and tensin homolog) is an upstream regulator of the PI3K/Akt/mTOR pathway, which can inhibit the activation of PI3K/Akt by converting PI (3,4,5)P3 (phosphatidylinositol phospholipid class 3 dephosphorylation) to PI(4,5)P2, causing dramatically higher levels of Arginase-1 and M2-subtype macrophage markers [61–63]. PI3K p110γ belongs to the class IB family of PI3K lipid kinases and controls immunosuppression via promoting macrophage polarization [64–66]. Recent research has demonstrated that PI3K p110γ reduces immunological responses, enhances pancreatic ductal adenocarcinoma cell invasion, and promotes transcriptional reprogramming of M2 macrophages [67]. The mTOR signaling pathway, a downstream effector of PI3K/Akt, is a crucial sensor of cellular trophic state and plays an important role in coordinating immune cells’ metabolism and inflammation, resulting in macrophage activation and polarization [64, 68–70]. Based on the above-mentioned evidence, we know that PI3K/Akt/mTOR signaling pathway is involved in regulating macrophage biology and polarization. Here, we demonstrated that exosomal miR-467b-3p derived from UTX^−/−^ ECs was uptake by macrophage and become a sink for PTEN, leading to the release of the repressive effect of PTEN on PI3K p110 and AKT, thereby promoting phosphorylation of mTOR signaling pathway. Thus, it is unsurprising that the UTX-miR-467b-3p-PTEN/PI3K/Akt/mTOR network regulates macrophage polarization in vitro.

In conclusion, this study reveals a mechanism underlying epigenetic modification-induced endothelial cell-macrophage crosstalk. This mechanism involves the UTX deletion epigenetically up-regulate miR-467b-3p in Tek^+^ ECs, and then miR-467b-3p transfer from ECs to macrophage by exosomes, decreasing PTEN expression and activating the PI3K/AKT/mTOR signaling pathway in macrophage, finally leading to polarizing macrophage to M2 subtype.

## Supplementary Information

**Additional file 1:** **Figure S1. **Verification of macrophage depletion in UTX^−/−^ mice after SCI. Immunofluorescence analysis of the efficacy of macrophage (Green) depletion in the injured site in sham, Clodronate liposomes and PBS liposomes groups on day 14 post-SCI. Iba-1^+^ microglia (red). Scale bar, 100 μm. **Figure S2. **Identification of endothelial cells and bone marrow-derived macrophages. (A) Representative morphological map of ECs. Scale bar, 20 μm. (B) Representative immunofluorescence images of CD31^+^ ECs (green) and DAPI (blue) staining. Red arrows: tight junctions between cells. Scale bars, 20 μm and 4 μm. (C) Representative flow cytometric Histogram of ECs with surface marker CD31. (D) Representative immunofluorescence images of bone marrow-derived macrophages stained for F4/80 (green) and CD11b (red). Scale bars, 20 μm and 5 μm. (E–F) Representative flow cytometric Histogram plots of bone marrow-derived macrophage surface markers for F4/80 and CD11b. n = 3/each group. **Figure S3.** Characterization of EC-derived exosomes and tracer experiments in vitro and in vivo. (A) Scanning electron microscope view of exosome morphology. Red arrows: spherical structures as well as characteristic bilayer-like structures. Scale bar, 100 nm. (B) Nanoparticle tracking analysis (NTA) to observe exosome size and distribution range. (C) Western blotting analysis of the expression levels of the characteristic proteins CD63 and TSG101 in exosomes and the expression level of the characteristic cell membrane protein Calnexin. (D) Immunofluorescence analysis of macrophage uptake of Dil-labeled EC-Exos (red). Scale bar, 20 μm. (E) Immunofluorescence staining of the macrophages taken up the Dil-labeled exosomes (red) observed by laser scanning confocal microscopy. Scale bar, 1 μm. (F) Immunofluorescence analysis of macrophages (green) taken up Dil-labeled EC-Exos (red) in the injured epicenter at 14 days after SCI. Scale bars, 50 μm and 20 μm. **Figure S4. **UTX^−/−^ ECs may not impact the microglia polarization. (A) Representative scatter plots of microglia phenotypes in sham, UTX^f/f^ mice, and UTX^−/−^ mice at 7 days after SCI. Cells were immunolabeled with CD45, CD11b, F4/80, CD206, and CD11c. Microglia are defined as CD45^low^CD11b^+^, activated state microglia are defined as F4/80^−^ in the CD45^low^CD11b^+^ gate, M1 microglia (CD11c^+^CD206^−^) and M2-type microglia (CD11c^−^CD206^+^). The numbers in the gates refer to the percentage of positive cells for each marker. (B) The percentage of microglia (CD45^low^CD11b^+^) in the total cells in (A). (C) The percentage of F4/80^−^ cells in the CD45^low^CD11b^+^ gate (microglia) in (A). (D-E) The percentage of M1 microglia (CD11c^+^CD206^−^) and M2 microglia (CD11c^−^CD206^+^) cells in the CD45^low^CD11b^+^F4/80^−^ gate (activated state microglia) in (A). (F-K) qRT-PCR analysis of the mRNA expression of M1 markers (iNOS2, TNF-ɑ, and CD86) and M2 markers (Arg-1, IL-10, and CD206) of LPS-stimulated macrophage when exposed to the Vehicle, UTX^f/f^-Exos, and UTX^−/−^-Exos. ^ns^*P* > 0.05, *P < 0.05, **P < 0.01 vs*.* corresponding control groups. n = 5/per group. **Figure S5. **Bioinformation analysis of the predicted potential target proteins of miR-467b-3p. (A) Overall plot of GO and KEGG enrichment analysis of the overlapped predicted potential target proteins of miR-467b-3p. (B) GO enrichment analysis of the overlapped predicted potential target proteins of miR-467b-3p. **Figure S6. **The regulatory relationship between UTX and H3K27me3/me2, miR-467b-3p, and PTEN in vitro and in vivo. (A) Western blotting analysis of the expression levels of H3K27me3/me2 in UTX^f/f^ ECs and UTX^−/−^ ECs. (B) Statistical analysis of H3K27me3/me2 expression in each group in (A). (C) qRT-PCR analysis of the miRNA-467b-3p expression in UTX^f/f^ sham mice, and UTX^f/f^ mice, UTX^−/−^ mice and UTX^−/−^ + UTX OE mice on day 7 after SCI. (D) Western blotting analysis of the PTEN expression levels in LPS-stimulated macrophages after UTX^f/f^-Exos, UTX^−/−^ Exos, and UTX^−/−^ + UTX OE-Exos treatment. (E) Statistical analysis of PTEN expression in each group in (D). (F) qRT-PCR analysis of the PTEN expression in LPS-stimulated macrophages after being treated with UTX^f/f^-Exos, UTX^−/−^ Exos, and UTX^−/−^ + UTX OE-Exos. **P < 0.01 vs. corresponding control groups. n = 5/per group. **Table S1.** Antibody Catalog. **Table S2.** All primer sequences used for qRT-PCR. **Table S3**. miRNA mimic/inhibitor sequences.

## Data Availability

The datasets used and/or analysed during the current study are available from the corresponding author on reasonable request.
