# Supplementary material for: UTX deletion promotes M2 macrophage polarization by epigenetically regulating endothelial cell-macrophage crosstalk after spinal cord injury
Source: J Nanobiotechnology. 2023 Jul 15;21:225. doi: 10.1186/s12951-023-01986-0 (PMC10350278; doi:10.1186/s12951-023-01986-0)
Supplement: Supplementary file 1 — Additional file 1: Figure S1. Verification of macrophage depletion in UTX−/− mice after SCI. Immunofluorescence analysis of the efficacy of macrophage (Green) depletion in the injured site in sham, Clodronate liposomes and PBS liposomes groups on day 14 post-SCI. Iba-1+ microglia (red). Scale bar, 100 μm. Figure S2. Identification of endothelial cells and bone marrow-derived macrophages. (A) Representative morphological map of ECs. Scale bar, 20 μm. (B) Representative immunofluorescence images of CD31+ ECs (green) and DAPI (blue) staining. Red arrows: tight junctions between cells. Scale bars, 20 μm and 4 μm. (C) Representative flow cytometric Histogram of ECs with surface marker CD31. (D) Representative immunofluorescence images of bone marrow-derived macrophages stained for F4/80 (green) and CD11b (red). Scale bars, 20 μm and 5 μm. (E–F) Representative flow cytometric Histogram plots of bone marrow-derived macrophage surface markers for F4/80 and CD11b. n = 3/each group. Figure S3. Characterization of EC-derived exosomes and tracer experiments in vitro and in vivo. (A) Scanning electron microscope view of exosome morphology. Red arrows: spherical structures as well as characteristic bilayer-like structures. Scale bar, 100 nm. (B) Nanoparticle tracking analysis (NTA) to observe exosome size and distribution range. (C) Western blotting analysis of the expression levels of the characteristic proteins CD63 and TSG101 in exosomes and the expression level of the characteristic cell membrane protein Calnexin. (D) Immunofluorescence analysis of macrophage uptake of Dil-labeled EC-Exos (red). Scale bar, 20 μm. (E) Immunofluorescence staining of the macrophages taken up the Dil-labeled exosomes (red) observed by laser scanning confocal microscopy. Scale bar, 1 μm. (F) Immunofluorescence analysis of macrophages (green) taken up Dil-labeled EC-Exos (red) in the injured epicenter at 14 days after SCI. Scale bars, 50 μm and 20 μm. Figure S4. UTX−/− ECs may not impact the microg [file 12951_2023_1986_MOESM1_ESM.docx]

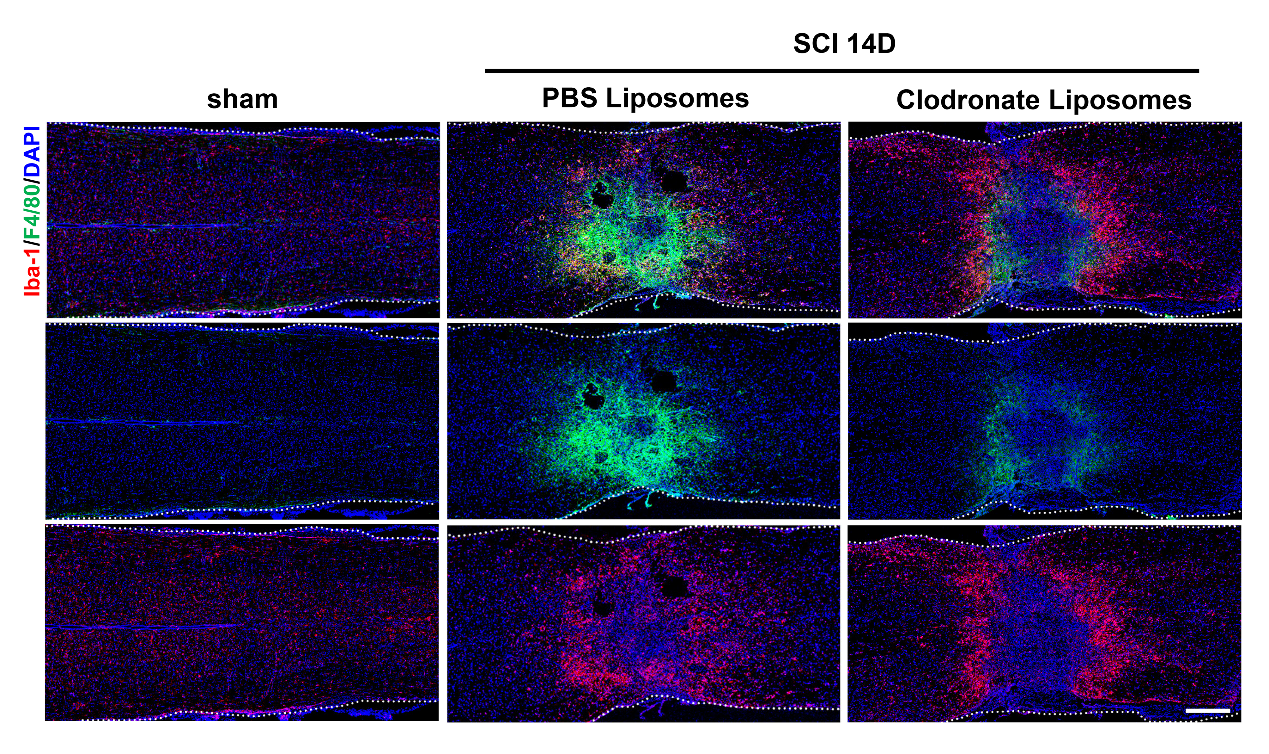


**Figure S1 Verification of macrophage depletion in UTX^-/-^ mice after SCI.** Immunofluorescence analysis of the efficacy of macrophage (Green) depletion in the injured site in sham, Clodronate liposomes and PBS liposomes groups on day 14 post-SCI. Iba-1^+^ microglia (red). Scale bar, 100μm.


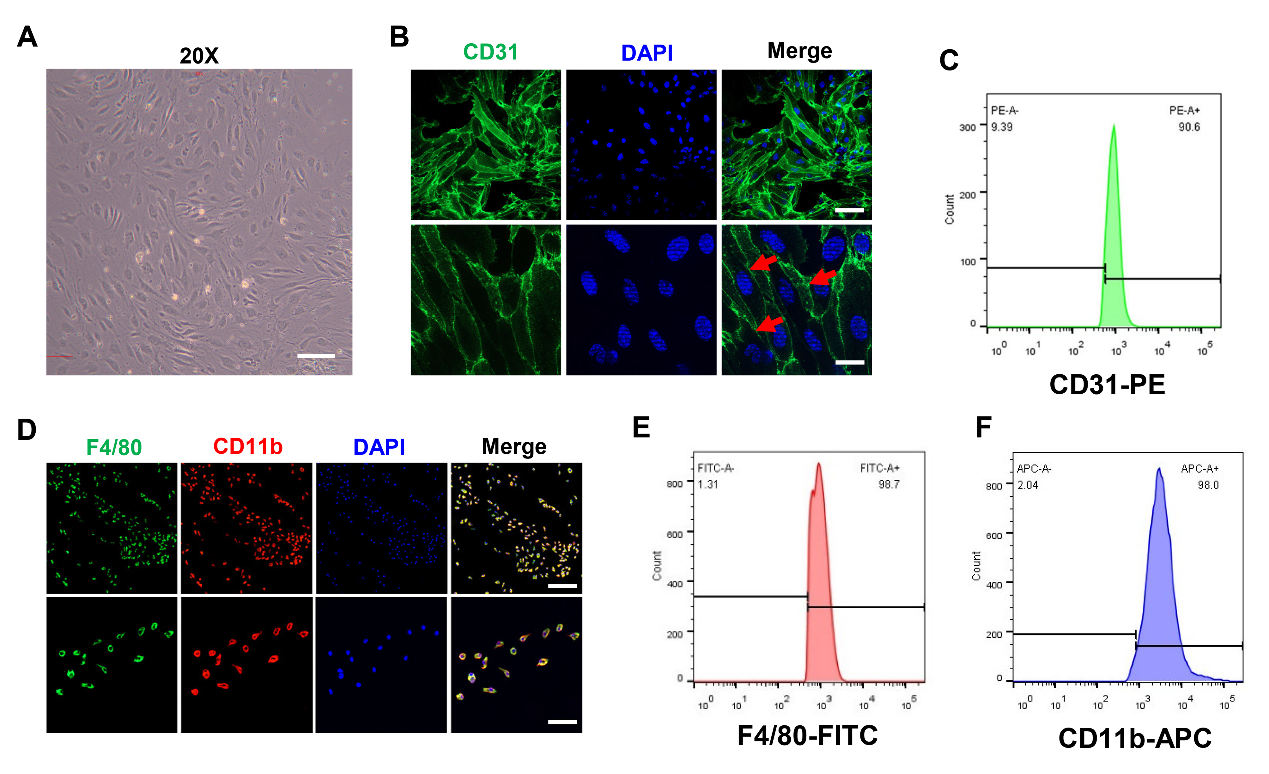


**Figure S2.** **Identification of endothelial cells and bone marrow-derived macrophages.** (A) Representative morphological map of ECs. Scale bar, 20μm. (B) Representative immunofluorescence images of CD31^+^ ECs (green) and DAPI (blue) staining. Red arrows: tight junctions between cells. Scale bars, 20μm and 4μm. (C) Representative flow cytometric Histogram of ECs with surface marker CD31. (D) Representative immunofluorescence images of bone marrow-derived macrophages stained for F4/80 (green) and CD11b (red). Scale bars, 20μm and 5μm. (E-F) Representative flow cytometric Histogram plots of bone marrow-derived macrophage surface markers for F4/80 and CD11b. n=3/each group.


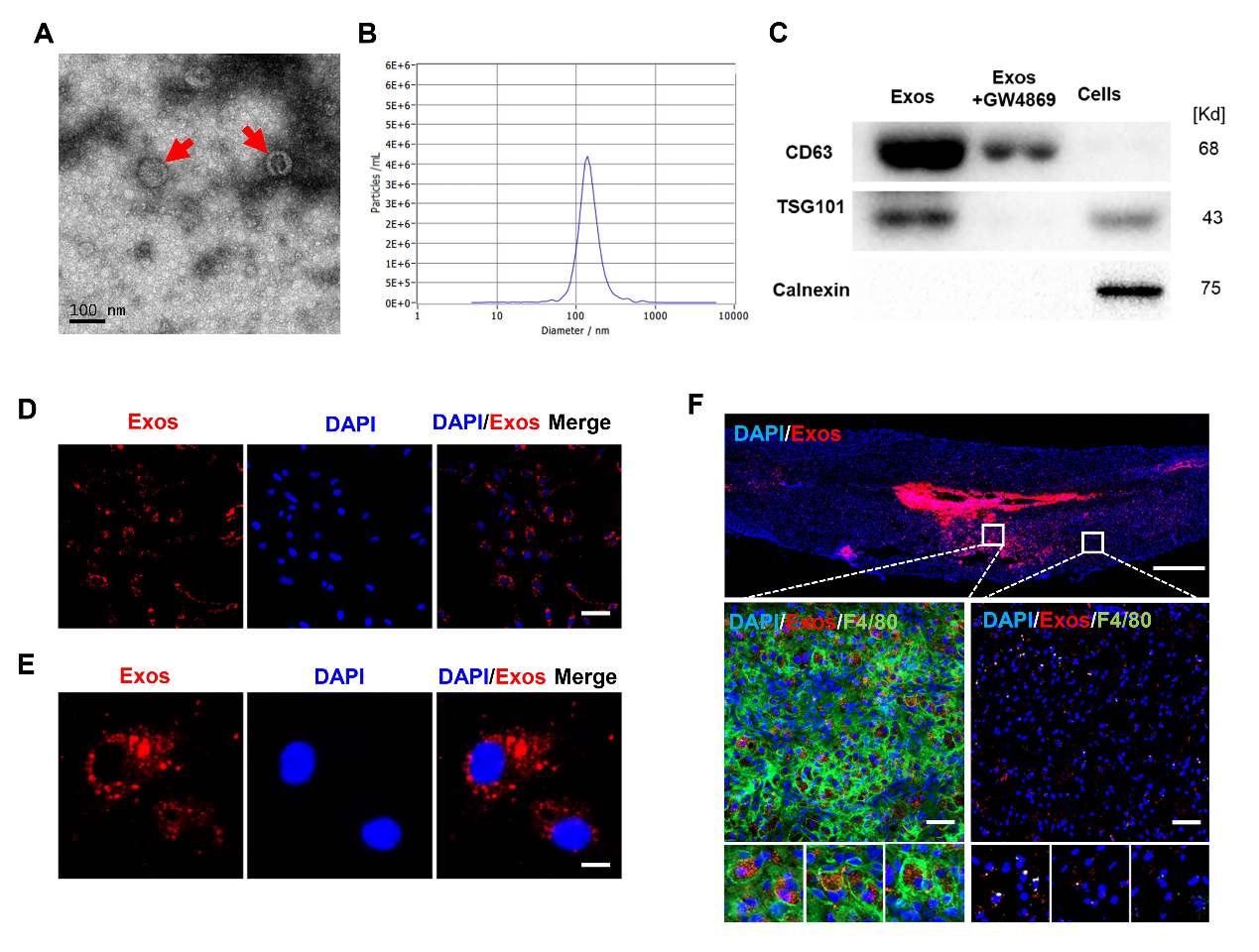


**Figure S3.** **Characterization of EC-derived exosomes and tracer experiments *in vitro* and *in vivo*.** (A) Scanning electron microscope view of exosome morphology. Red arrows: spherical structures as well as characteristic bilayer-like structures. Scale bar, 100 nm. (B) Nanoparticle tracking analysis (NTA) to observe exosome size and distribution range. (C) Western blotting analysis of the expression levels of the characteristic proteins CD63 and TSG101 in exosomes and the expression level of the characteristic cell membrane protein Calnexin. (D) Immunofluorescence analysis of macrophage uptake of Dil-labeled EC-Exos (red). Scale bar, 20μm. (E) Immunofluorescence staining of the macrophages taken up the Dil-labeled exosomes (red) observed by laser scanning confocal microscopy. Scale bar, 1μm. (F) Immunofluorescence analysis of macrophages (green) taken up Dil-labeled EC-Exos (red) in the injured epicenter at 14 days after SCI. Scale bars, 50μm and 20μm.


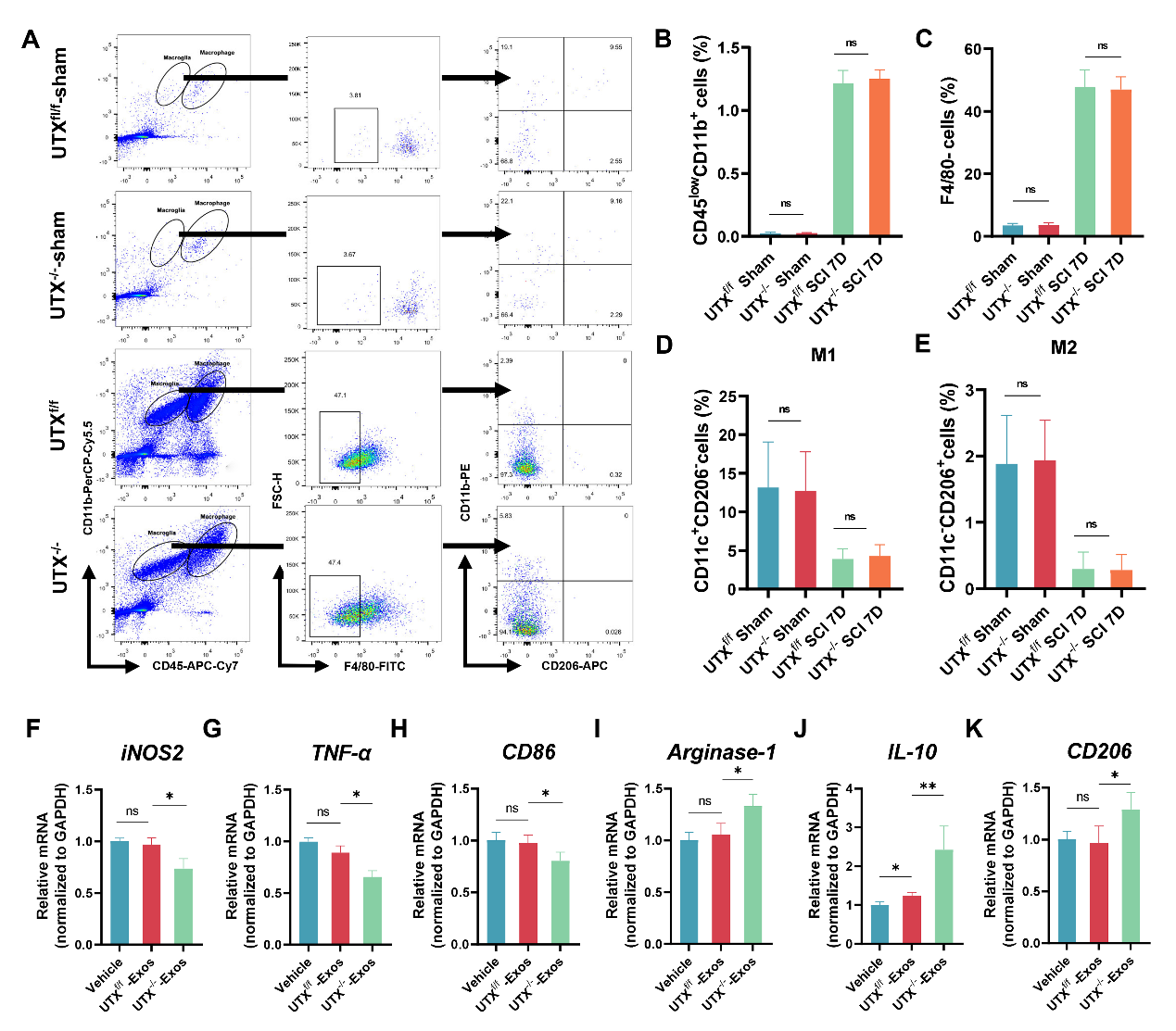


**Figure S4.** **UTX^-/-^ ECs may not impact the microglia polarization.** (A) Representative scatter plots of microglia phenotypes in sham, UTX^f/f^ mice, and UTX^-/-^ mice at 7 days after SCI. Cells were immunolabeled with CD45, CD11b, F4/80, CD206, and CD11c. Microglia are defined as CD45^low^CD11b^+^, activated state microglia are defined as F4/80^-^ in the CD45^low^CD11b^+^ gate, M1 microglia (CD11c^+^CD206^-^) and M2-type microglia (CD11c^-^CD206^+^). The numbers in the gates refer to the percentage of positive cells for each marker. (B) The percentage of microglia (CD45^low^CD11b^+^) in the total cells in (A). (C) The percentage of F4/80^-^ cells in the CD45^low^CD11b^+^ gate (microglia) in (A). (D-E) The percentage of M1 microglia (CD11c^+^CD206^-^) and M2 microglia (CD11c^-^CD206^+^) cells in the CD45^low^CD11b^+^F4/80^-^ gate (activated state microglia) in (A). (F-K) qRT-PCR analysis of the mRNA expression of M1 markers (iNOS2, TNF-ɑ, and CD86) and M2 markers (Arg-1, IL-10, and CD206) of LPS-stimulated macrophage when exposed to the Vehicle, UTX^f/f^-Exos, and UTX^-/-^-Exos. ^ns^*P*>0.05, **P*<0.05, ***P*<0.01 *Vs.* corresponding control groups. n=5/per group.


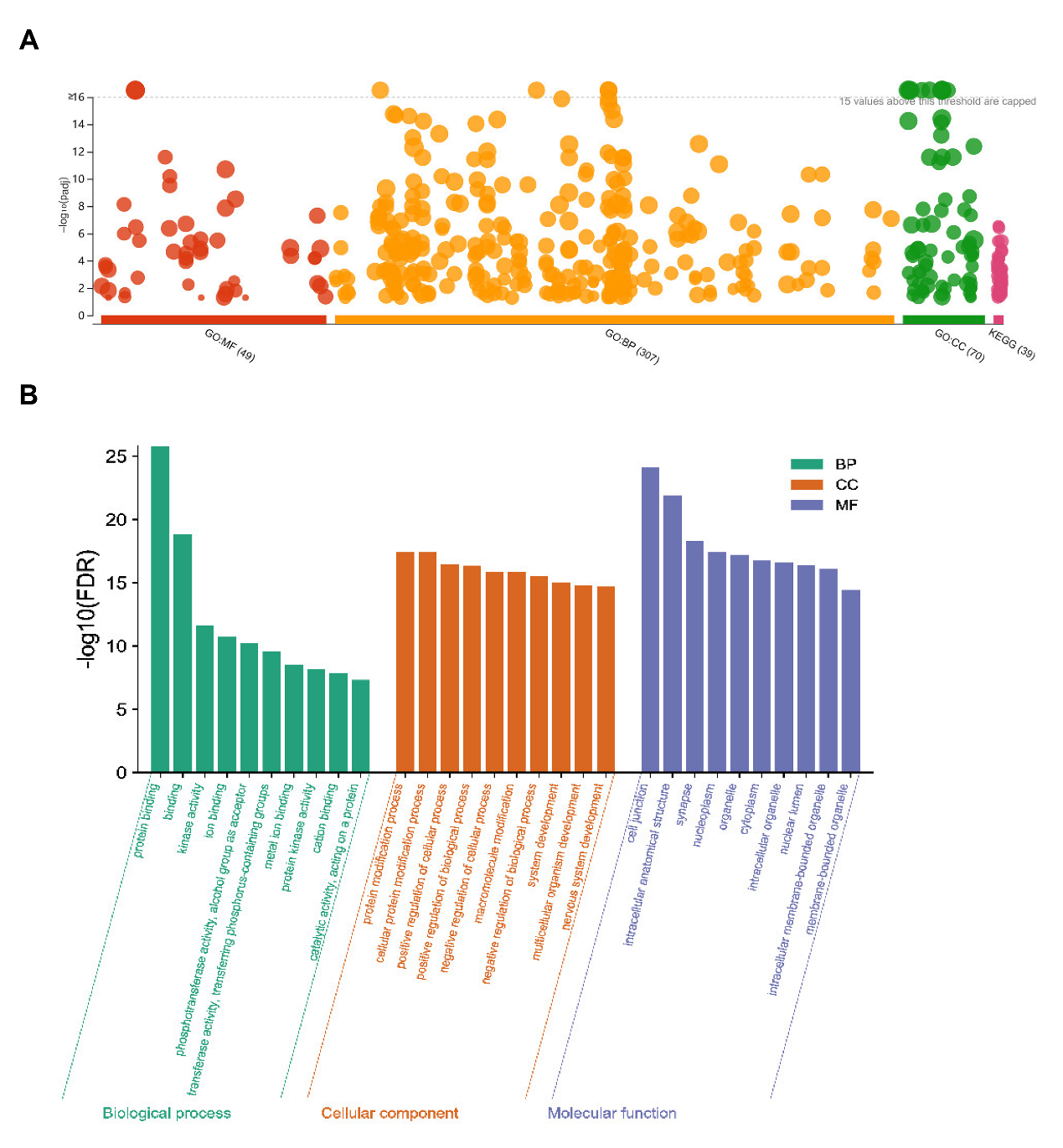


**Figure S5. Bioinformation analysis of the predicted potential target proteins of miR-467b-3p.** (A) Overall plot of GO and KEGG enrichment analysis of the overlapped predicted potential target proteins of miR-467b-3p. (B) GO enrichment analysis of the overlapped predicted potential target proteins of miR-467b-3p.


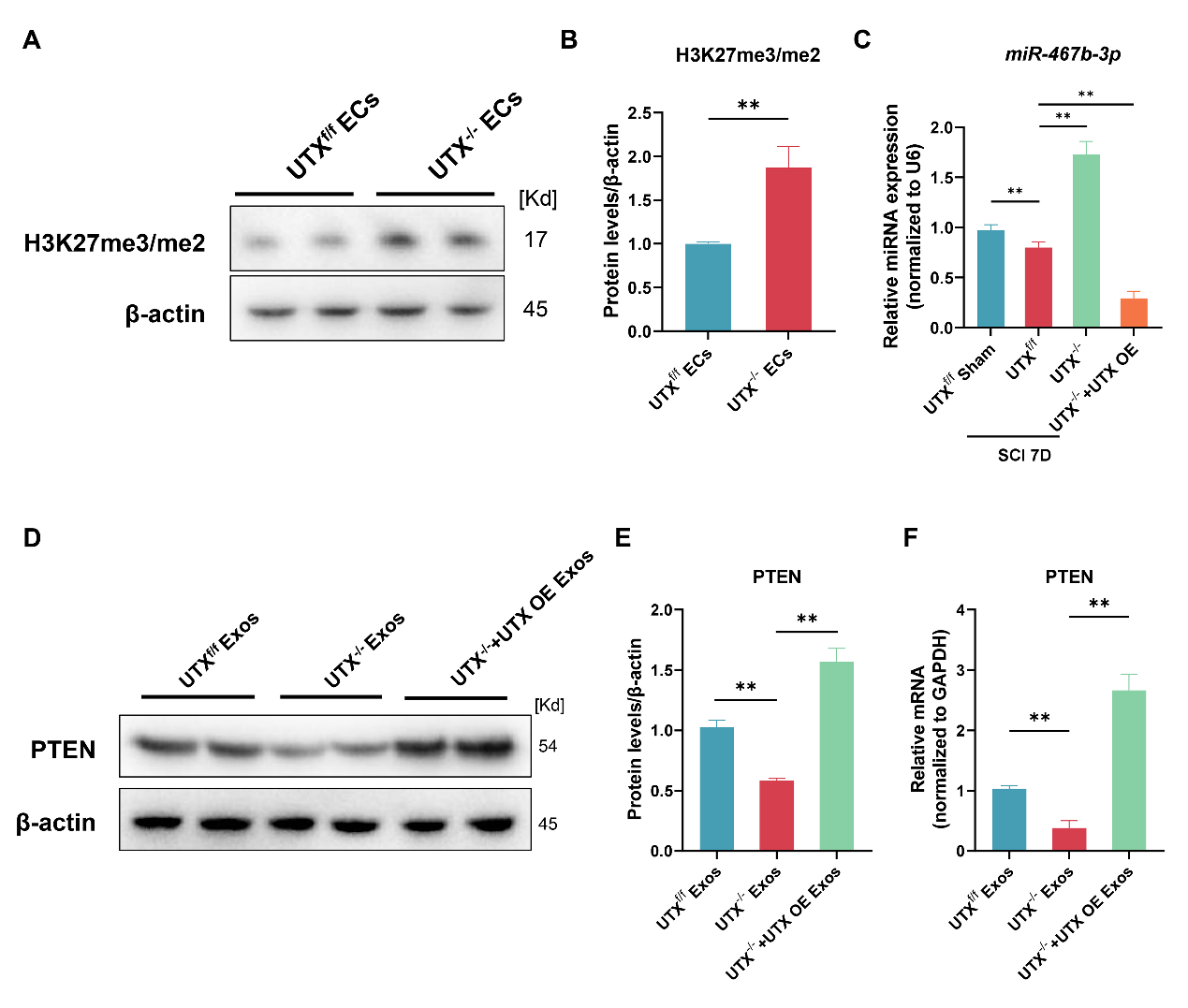


**Figure S6. The regulatory relationship between UTX and H3K27me3/me2, miR-467b-3p, and PTEN in vitro and in vivo.**(A) Western blotting analysis of the expression levels of H3K27me3/me2 in UTX^f/f^ ECs and UTX^-/-^ ECs. (B) Statistical analysis of H3K27me3/me2 expression in each group in (A). (C) qRT-PCR analysis of the miRNA-467b-3p expression in UTX^f/f^ sham mice, and UTX^f/f^ mice, UTX^-/-^ mice and UTX^-/-^+UTX OE mice on day 7 after SCI. (D) Western blotting analysis of the PTEN expression levels in LPS-stimulated macrophages after UTX^f/f^-Exos, UTX^-/-^ Exos, and UTX^-/-^+UTX OE-Exos treatment. (E) Statistical analysis of PTEN expression in each group in (D). (F) qRT-PCR analysis of the PTEN expression in LPS-stimulated macrophages after being treated with UTX^f/f^-Exos, UTX^-/-^ Exos, and UTX^-/-^+UTX OE-Exos. **P<0.01 Vs. corresponding control groups. n=5/per group.


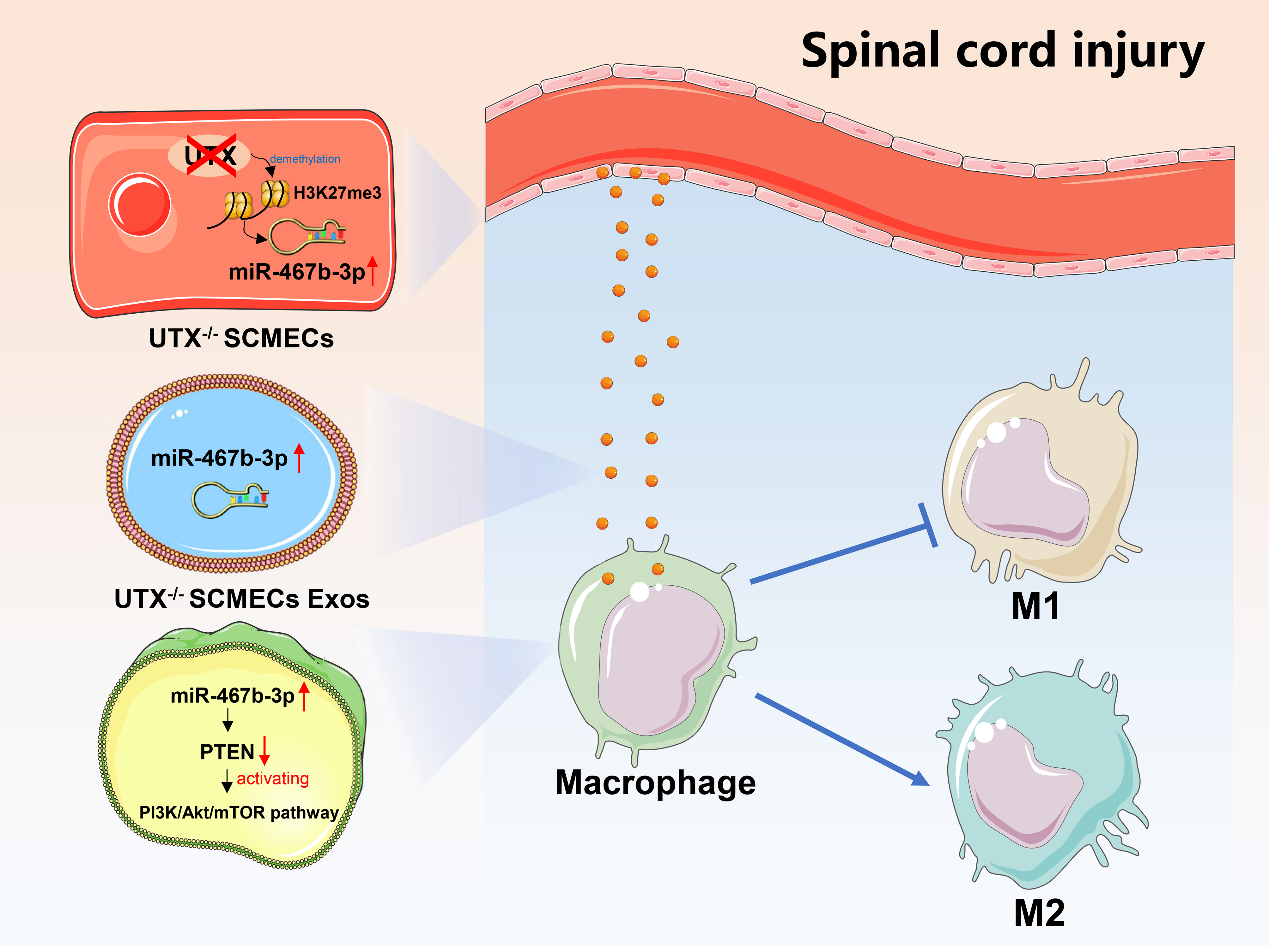


**Graphic Abstract**

Histone demethylase UTX deletion in Tek^+^ ECs upregulates the miR-467b-3p expression, which transfers to macrophages by exosomes and activates the PI3K/AKT/mTOR signaling by decreasing PTEN expression, finally polarizing macrophage to the M2 subtype and promoting the functional recovery after SCI.

Table S1 Antibody Catalog

| **IF antibody** | **company** | **catalog number** | **dilution** |
| --- | --- | --- | --- |
| goat anti-CD31 | R&D Systems | FAB3628G-100 | 1:400 |
| rabbit anti-UTX | Millipore | ABE409 | 1:200 |
| rat anti-F4/80 | Abcam | ab6640 | 1:100 |
| rabbit anti-CD11b | Abcam | ab133357 | 1:200 |
| goat anti-CD206 | R&D Systems | AF2535 | 1:100 |
| rabbit anti-iNOS | GeneTex | GTX130246 | 1:100 |
| rabbit anti-CD86 | CST | 91882 | 1:100 |
| rabbit anti-Iba1 | WAKO | 019-19741 | 1:200 |
| **WB antibody** | **company** | **catalog number** | **dilution** |
| rabbit anti-Calnexin | Abcam | ab22595 | 1:1000 |
| rabbit anti-CD63 | Abcam | ab134045 | 1:1000 |
| rabbit anti-TSG101 | Abcam | ab125011 | 1:1000 |
| rabbit anti-iNOS | GeneTex | GTX130246 | 1:1000 |
| mouse anti-Arginase 1 | Santa Cruz | sc-271430 | 1:1000 |
| rabbit anti-β-actin | CST | 4970 | 1:5000 |
| rabbit Anti-PTEN | CST | 9188 | 1:1000 |
| rabbit Anti-AKT | CST | 4691 | 1:1000 |
| rabbit Anti-p-AKT | CST | 4060 | 1:1000 |
| rabbit Anti-mTOR | CST | 2983 | 1:1000 |
| rabbit Anti-p-mTOR | CST | 5536 | 1:1000 |
| rabbit Anti-PI3K 110γ | CST | 5405 | 1:1000 |
| rabbit anti-Tri-Methyl-Histone 3 | CST | 9733 | 1:1000 |
| **Flow cytometry antibody** | **company** | **catalog number** | **dilution** |
| CD11b-PerCP-Cy5.5 | Biolegend | 101227 | 1:200 |
| CD45-APC-Cy7 | Biolegend | 157203 | 1:200 |
| F4/80-FITC | Biolegend | 157310 | 1:200 |
| CD11c-PE | Biolegend | 117307 | 1:200 |
| CD206-APC | Biolegend | 141708 | 1:200 |
| DAPI | BD Bioscience | 564907 | 1:5000 |

Table S2 All primer sequences used for qRT-PCR.

| *iNOS2* | Forward | ACATCGACCCGTCCACAGTAT |
| --- | --- | --- |
|  | Reverse | CAGAGGGGTAGGCTTGTCTC |
| *TNF-α* | Forward | CAGGCGGTGCCTATGTCTC |
|  | Reverse | CGATCACCCCGAAGTTCAGTAG |
| *CD86* | Forward | TGTTTCCGTGGAGACGCAAG |
|  | Reverse | TTGAGCCTTTGTAAATGGGCA |
| *CD38* | Forward | ACCACGAAGCACTTTTCTGAC |
|  | Reverse | GGCGTAGTCTTCTCTTGTGATG |
| *Arginase-1* | Forward | CTCCAAGCCAAAGTCCTTAGAG |
|  | Reverse | GGAGCTGTCATTAGGGACATCA |
| *CD206* | Forward | CTCTGTTCAGCTATTGGACGC |
|  | Reverse | TGGCACTCCCAAACATAATTTGA |
| *IL-10* | Forward | GCTCTTACTGACTGGCATGAG |
|  | Reverse | CGCAGCTCTAGGAGCATGTG |
| *Ym-1* | Forward | CAGGTCTGGCAATTCTTCTGAA |
|  | Reverse | GTCTTGCTCATGTGTGTAAGTGA |
| *GAPDH* | Forward | AGGTCGGTGTGAACGGATTTG |
|  | Reverse | TGTAGACCATGTAGTTGAGGTCA |
| *PTEN* | Forward | TGGATTCGACTTAGACTTGACCT |
|  | Reverse | GCGGTGTCATAATGTCTCTCAG |
| *AKT-2* | Forward | ACGTGGTGAATACATCAAGACC |
|  | Reverse | GCTACAGAGAAATTGTTCAGGGG |
| *AKT-3* | Forward | TGGGTTCAGAAGAGGGGAGAA |
|  | Reverse | AGGGGATAAGGTAAGTCCACATC |
| *STAT-1* | Forward | TCACAGTGGTTCGAGCTTCAG |
|  | Reverse | GCAAACGAGACATCATAGGCA |
| *STAT-4* | Forward | TGGCAACAATTCTGCTTCAAAAC |
|  | Reverse | GAGGTCCCTGGATAGGCATGT |
| *PGC-1α* | Forward | TATGGAGTGACATAGAGTGTGCT |
|  | Reverse | CCACTTCAATCCACCCAGAAAG |
| *PIK3R1* | Forward | ACACCACGGTTTGGACTATGG |
|  | Reverse | GGCTACAGTAGTGGGCTTGG |
| *IRF-1* | Forward | ATGCCAATCACTCGAATGCG |
|  | Reverse | TTGTATCGGCCTGTGTGAATG |
| *JAK-2* | Forward | TTGTGGTATTACGCCTGTGTATC |
|  | Reverse | ATGCCTGGTTGACTCGTCTAT |

Table S3 miRNA mimic/inhibitor sequences

| *miRNA-467b-3p mimic* | sense primer | AUAUACAUACACACACCAACAC |
| --- | --- | --- |
|  | antisense primer | GUGUUGGUGUGUGUAUGUAUAU |
| *inhibitor* | antisense primer | GUGUUGGUGUGUGUAUGUAUAU |
| miRNA-7224-3p mimic | sense primer | UCCACUGAGAGGACCACCCAC |
|  | antisense primer | GUGGGUGGUCCUCUCAGUGGA |
| inhibitor | antisense primer | GUGGGUGGUCCUCUCAGUGGA |
| miRNA-346-5p mimic | sense primer | AGGCAGGGGCUGGGCCUGCAGC |
|  | antisense primer | GCUGCAGGCCCAGCCCCUGCCU |
| inhibitor | antisense primer | GCUGCAGGCCCAGCCCCUGCCU |
| miRNA-466l-3p mimic | sense primer | UAUAAAUACAUGCACACAUAUU |
|  | antisense primer | AAUAUGUGUGCAUGUAUUUAUA |
| inhibitor | antisense primer | AAUAUGUGUGCAUGUAUUUAUA |
| miRNA mimic Negative control | sense primer | UUUGUACUACACAAAAGUACUG |
|  | antisense primer | CAGUACUUUUGUGUAGUACAAA |
| inhibitor Negative control | antisense primer | CAGUACUUUUGUGUAGUACAAA |
